# Prenatal Exposure to Dibutyl Phthalate and Its Negative Health Effects on Offspring: In Vivo and Epidemiological Studies

**DOI:** 10.3390/jox14040109

**Published:** 2024-12-19

**Authors:** Ana R. Quelhas, Melissa Mariana, Elisa Cairrao

**Affiliations:** 1 Health Sciences Research Centre (CICS-UBI), University of Beira Interior, 6200-506 Covilhã, Portugal; ana.rita.quelhas@ubi.pt (A.R.Q.); melissa.r.mariana@gmail.com (M.M.); 2 Faculty of Sciences (FC-UBI), University of Beira Interior, 6201-001 Covilhã, Portugal; 3 Faculty of Health Sciences (FCS-UBI), University of Beira Interior, 6200-506 Covilhã, Portugal

**Keywords:** phthalate, plastic contaminants, endocrine disruptor, pregnancy, maternal–fetal exposure

## Abstract

Dibutyl phthalate (DBP) is a low-molecular-weight phthalate commonly found in personal care products, such as perfumes, aftershaves, and nail care items, as well as in children’s toys, pharmaceuticals, and food products. It is used to improve flexibility, make polymer products soft and malleable, and as solvents and stabilizers in personal care products. Pregnancy represents a critical period during which both the mother and the developing embryo can be significantly impacted by exposure to endocrine disruptors. This article aims to elucidate the effects of prenatal exposure to DBP on the health and development of offspring, particularly on the reproductive, neurological, metabolic, renal, and digestive systems. Extensive research has examined the effects of DBP on the male reproductive system, where exposure is linked to decreased testosterone levels, reduced anogenital distance, and male infertility. In terms of the female reproductive system, DBP has been shown to elevate serum estradiol and progesterone levels, potentially compromising egg quality. Furthermore, exposure to this phthalate adversely affects neurodevelopment and is associated with obesity, metabolic disorders, and conditions such as hypospadias. These findings highlight how urgently stronger laws prohibiting the use of phthalates during pregnancy are needed to lower the risks to the fetus’s health and the child’s development.

## 1. Introduction

An endocrine disruptor is “an agent that interferes with the synthesis, secretion, transport, binding or elimination of natural hormones in the body that is responsible for maintaining homeostasis, reproduction, development and/or behavior”, according to the US Environmental Protection Agency (EPA) [1]. Similarly, the World Health Organization (WHO) describes an endocrine disruptor as “an exogenous substance or mixture that alters function(s) of the endocrine system and consequently causes adverse health effects in an intact organism, or its progeny, or (sub)populations” [2]. Thus, endocrine disruptors alter endocrine functions by mimicking natural hormones, such as estrogens, androgens or thyroid hormones, or alter the hormones’ metabolism by blocking or antagonizing their interaction with receptors [3].

Phthalates, considered as endocrine disruptors, are phthalic acid diesters and synthetic organic chemicals classified into high and low molecular weight [3,4]. High-molecular-weight phthalates are used in polyvinyl chloride (PVC) polymers and plasticizer applications, plastics, food packaging and food processing materials, and vinyl toys, among others [4]. Di(2-ethylhexyl) phthalate (DEHP) is the most widely used and studied high-molecular-weight phthalate. Low-molecular-weight phthalates are used in personal care products, paints, adhesives and enteric-coated tablets [4]. Dibutyl phthalate (DBP), which is the most widely studied low-molecular-weight phthalate, has also been found in personal care products (perfumes, aftershaves, nail care products) and in children’s toys, pharmaceuticals, food products, among others, improving flexibility and making polymeric products soft and malleable [4,5,6]. It was also used as an insecticide and acaricide [7,8]. However, considering the adverse effects demonstrated for DBP, this compound has been prohibited in cosmetic and childcare products [9,10,11].

Phthalates are easily released into the environment as they are not covalently bound to plastic [12]. Thus, humans are exposed to various types of phthalates through ingestion, inhalation and dermal exposure throughout their lives, including intrauterine life, since these compounds can cross the placental barrier [4,13]. With regard to their metabolism, phthalates are rapidly metabolized into their respective monoesters through phase I biotransformation reactions. Specifically, DBP is hydrolyzed and transformed into monobutyl phthalate (MBP), its main metabolite. It is subsequently excreted in the urine, usually after glucuronidation [13]. Thus, this compound has already been found in biological matrices in the general population, including urine [14,15,16], serum [17], semen [18], nails [19], and also in maternal urine [20,21,22], maternal serum [23], breast milk [4,24] and amniotic fluid [25].

Pregnancy is a sensitive period in which the mother and the embryo can be simultaneously affected by exposure to endocrine disruptors [3]. DBP is a phthalate known for its estrogenic and anti-androgenic activity in vitro, and is recognized as an environmental estrogen [26]. However, its effect at the prenatal level has been little explored, and the use of these products by pregnant women is a worrying reality. Therefore, the articles in this review will be categorized by the reproductive, neurological, metabolic, renal, and digestive alterations that DBP prenatal exposure may cause in offspring.

## 2. Approach to the Review

**Figure 1:**
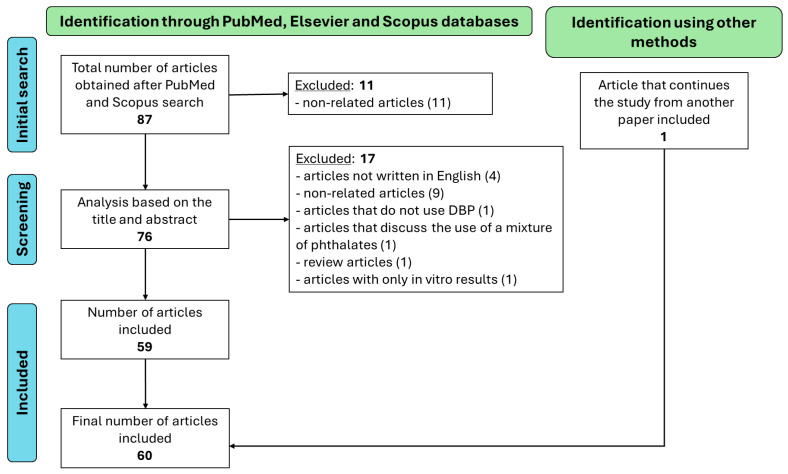
Flow diagram of the literature review process.

In this review, experimental studies evaluating the effects of prenatal exposure to DBP on cells, animal models and humans are summarized. The literature review was carried out using articles available in PubMed, Elsevier and Scopus databases using the “AND” and “OR” Boolean operators and “dibutyl phthalate” as the MeSH Major Topic. Terms related to prenatal exposure and pregnancy were used, such as “prenatal exposure delayed effects”, “maternal exposure” and “pregnancy”. At the end of the literature review, 87 articles were collected in October 2023, but only 61 papers are included in this review (Figure 1).

The inclusion criteria were as follows: (1) original articles; (2) studies carried out in animal models, in vitro or using epidemiological data; (3) studies with a control group; (4) articles that clearly differentiated the results between the different phthalates; (5) articles from the last 10 years; (6) articles that continue a study from another paper included. The exclusion criteria applied were as follows: (1) non-original articles, such as reviews; (2) studies using mixtures of phthalates; (3) articles not written in English; (4) duplicate articles; (5) articles with restricted access; (6) in vitro articles.

## 3. In Vivo Studies

The in vivo studies analyzed in this review on the effects of prenatal exposure to DBP were mainly carried out on rats, namely, Sprague Dawley, C57BL/6 mice, Wistar, CD1 mice, a/a mice (longitudinal model), BALB/c mice and Mongolian gerbils. However, a study was also carried out on White Leghorn chick eggs. The effects of DBP on the neurological, metabolic, renal, digestive, and reproductive systems, along with the design parameters of each study outlined, are presented in Table 1 and Table 2. These tables summarize key information, including the duration of exposure, dosage, route of exposure, solvent utilized, and the main conclusions of the studies. A detailed discussion of these findings will follow.

### 3.1. Nervous System

Currently, there are very few published articles studying the nervous system in vivo, via neurotoxicity and maternal behavior. The embryonic period and the perinatal period in rats are phases of structural and functional development of the hypothalamus. For these animals, sex-specific differentiation in brain regions such as the hypothalamus depends on steroids exposure [27]. On the other hand, aromatase (AROM), an enzyme responsible for transforming androgens into estrogens, also plays an important role in neuroprotection. Estrogen receptor β (ER-β) and brain-derived neurotrophic factor (BDNF) regulate synaptic plasticity in the hippocampus, and the former also improves memory. The cAMP response element binding protein (CREB)-BNDF signaling pathway is activated during neuroprotective functions mediated by the ER [26]. Regarding the role of astrocytes, which are the most abundant glial cells in the central nervous system (CNS), they respond to injuries upon activation. In the event of serious injury, the activated astrocytes proliferate excessively, forming glial scars at the damaged site. STAT3 is a transcription factor that regulates astrocyte activation and is activated by the phosphorylation of Janus kinase (JAK), which in turn is activated by interleukin-6 (IL-6) [28]. In this sense, the following studies discussed are divided by rat species, since they have different morphological and physiological characteristics depending on the species.

Regarding studies on Sprague Dawley rats, Li et al. used female rats daily intragastrically exposed from gestational day (GD) 6 to postnatal day (PND) 21 [26]. In 2021, Hunter et al. also exposed this species orally, every two days, from GD14.5 to PND6 [27], and both studies used the same dose of DBP (500 mg/kg). The first study found that exposure to this phthalate increased estrogen in both immature offspring at 21 days of age and mature offspring at 60 days of age, and reduced testosterone in immature offspring and mature males. The immature offspring showed an increase in the expression of AROM and a decrease in the expressions of ER-β, BDNF and CREB in the hippocampus, while the mature offspring did not obtain significant results [26]. On the other hand, Hunter et al. observed a small reduction in body weight in the adult offspring and, on the 10th day after birth, the male pups showed a reduction in anogenital distance. As for hypothalamic gene expression at the beginning of puberty (PND24), the authors found that in female offspring, there was an increase in the expression of Esr2 and a reduction in the expression of Avp, genes involved in sexual or social behavior, although the result of the former was not statistically significant. There was also a reduction in the expression of the Gnrh1 gene, involved in the regulation of the hypothalamic–pituitary–adrenal axis; however, these effects did not persist into adulthood in the females. As for sexual behavior, in the male offspring, there was an increase in the latency period possibly due to transient erectile dysfunction, since it has been shown that penile erection in rats is linked to the hypothalamic expression of oxytocin, which was found to be slightly decreased in male rats. The female offspring, on the other hand, remained sexually receptive [27]. Both studies found that the effects did not persist into adulthood, and concluded that immature animals could recover over time and, consequently, DBP did not show long-term effects in these animals [26,27].

Neurodevelopment was studied by evaluating synaptic formation, astrocyte activation, and neuronal tissue [28,29,30]. Lee et al. used female C57BL/6 mice orally exposed to 50 and 100 mg/kg/day of DBP from GD13 until the end of lactation (PND15). The authors found a delay in the latency period; however, this occurred in females rather than males. In addition, the offspring showed decreased scores in motor development and memory tests. The cerebral cortex of the offspring prenatally exposed to DBP showed that the genes associated with the dopamine system were up-regulated, and they therefore concluded that this exposure could cause hyperactivity in the offspring and a delay in neurological development. On the other hand, mothers treated with DBP showed a negative regulation of transcription factors associated with neuronal plasticity and survival, as well as the genes that activate these factors, namely, BDNF, CREB and EFGR-1 [29]. These results are in agreement with those obtained by Li et al. [26]. Only one study addressed the BALB/c mice species, analyzing DBP exposure (50, 250 or 500 mg/kg/day intragastrically) from GD12.5 until birth (GD21.5) [28]. The results show increased astrocyte activation, increased STAT3 protein expression and decreased expressions of SOCS1 and SOCS3 (proteins that negatively regulate the JAK2/STAT3 pathway) in the hippocampus, showing that DBP exposure activated the JAK2/STAT3 signaling pathway in astrocytes. The authors concluded that this activation affected the morphology of dendrites and abnormally increased the synaptic formation of neurons in the hippocampus, since exposure to DBP promoted the appearance of immature dendrites and an increase in the number of dendrites in the CA3 region of the hippocampus. These effects may have resulted in the decreased autonomous and exploratory behavior of the offspring observed by the researchers [28]. Wistar rat species were only used by Radha and Basha, who orally subjected the rats to 500 mg/kg/day of DBP from GD6 to GD21 and during lactation. However, in this study, the rats were exposed continuously for three consecutive generations. Exposure to DBP decreased the activity of the antioxidant enzymes superoxide dismutase (SOD) and catalase (CAT) in the rats’ neuronal tissue, leading to oxidative stress by DNA damage through lipid peroxidation. Similarly, lipid peroxidation was also found to be elevated in the rats of the F3 generation in all the areas of neuronal tissue studied, namely, the cerebral cortex, cerebellum, hippocampus, medulla oblongata and spinal cord. Consequently, there was also a decrease in the content of nucleic acids in all areas of the brain and significant damage to DNA chains in all generations, with a higher incidence in the F3 generation. The cerebellum regions of the F1 and F2 generations showed a lower amount of RNA and DNA, while in the F3 generation, the hippocampus showed lower RNA levels, and the cerebellum and spinal cord had lower DNA levels. Thus, Radha and Basha (2022) concluded that oxidative stress induced by DBP caused DNA damage in specific regions of the rat brain over three generations [30].

From a different perspective, Wang et al. used eggs from the White Leghorn chicken species, into the yolk of which different concentrations of DBP (0.1–1000 µM) were directly injected [21]. All the DBP concentrations studied led to the development of neural tube defects, decreased body weight and increased malformations in the embryos. Besides this, embryos treated with 1 µM of DBP showed the poor development of the chorioallantoic vascular membrane. The authors also found that exposure to DBP increased apoptosis in embryos and oxidative stress levels, as SOD activity decreased and malondialdehyde (MDA) concentration increased. In this study, ferrous sulfate, folic acid or choline supplements were also used to analyze the possible reversion of the DBP’s effects on embryos. However, only a high concentration of choline (CHO, 100 µg/µL) protected the embryos from DBP-induced embryonic malformations and inhibited the development of oxidative stress [21].

Thus, all the in vivo studies analyzed showed that gestational exposure to DBP leads to the poor development of the offspring’s nervous system, promoting an increase in the latency period in both males and females, and a decrease in autonomous and exploratory behavior. However, Li et al. and Hunter et al. found that the animals were able to recover over time, while Radha and Basha observed that the DNA damage caused by DBP exposure remained over three generations. Comparing these studies is difficult due to the use of different animal species, DBP concentrations and exposure times; however, the results were consistent with each other, suggesting that DBP may have adverse effects on the nervous system.

### 3.2. Metabolism

Obesity is a metabolic disease characterized by an energy imbalance and excessive fat accumulation [31]. In addition to genetic factors, poor diet and a sedentary lifestyle, exposure to some endocrine disruptors has also been associated with the development of obesity and, for this reason, they have been known as “obesogens” [31,32]. In this sense, peroxisome proliferator-activated receptors (PPARs), nuclear receptors that control lipid metabolism and insulin sensitivity, mainly PPARγ, in peripheral tissues, have already been associated with these compounds [33]. Currently, the number of in vivo studies on the development of obesity and/or gestational diabetes mellitus (GDM) after perinatal exposure to DBP is still low.

Regarding the effect of this compound on obesity in 2018, Christante et al. and Negrin et al. evaluated exposure to DBP (100 mg/kg/day) from GD8 to GD23 in Mongolian gerbils [34,35]. The following year, Venturelli et al. used pregnant Wistar rats exposed to 5, 50 and 500 mg/kg/day of DBP, orally, during GD13–21 [33]. On the other hand, Neier et al. studied the impacts of exposure to this phthalate longitudinally, at the beginning (2 months) and at the end of adulthood (8 months). They used a/a mice exposed to 25 mg/kg/day of DBP orally from 2 weeks before mating until the PND21 [32]. In 2020, Li et al. used pregnant female SPF C57BL/6J mice exposed to 5 mg/kg/day of DBP, orally, from GD12 to PND7 [31]. Zhou et al. studied only the effects of perinatal exposure to DBP on glycolytic metabolism in female offspring, and observed the role of the PTEN/PI3K/AKT signaling pathway. For this, pregnant female Sprague-Dawley rats were exposed to 33, 66 and 132 mg/kg/day, intraperitoneally, from GD7 to PND21 [36].

Venturelli et al. [33], Li et al. [31], Zhou et al. [36] and Christante et al. [34] found an increase in the body weight of the F1 generation, and Neier et al. [32] observed an increase in the body weight of the female offspring. On the other hand, Wakui et al. [37,38], Chen et al. [39] and Okayama et al. [40] found that the body weight of male offspring was not affected by exposure to DBP. In 2018, Negrin et al. found that exposure to DBP led to increased serum levels of triglycerides, total cholesterol and non-HDL cholesterol [35].

Venturelli et al. observed that adult males in the group exposed to 5 mg/kg/day of DBP showed an increase in plasma cholesterol concentration. On the other hand, in the group exposed to 500 mg/kg/day of DBP, there was an increase in fasting plasma glucose concentration in both males and females, and an increase in triglyceride concentration only in females. In this group, the vaginal opening of the cubs was delayed, showing an anti-estrogenic effect of DBP, since vaginal opening depends on the increase in estradiol levels during puberty. These authors also found a decrease in the expression of pancreatic and duodenal homeobox-1 protein (PDX-1), which is important during the perinatal development of the pancreas and insulin regulation. Despite being non-significant, these effects were more prominent in males. In addition, there was also a non-significant reduction in the expression of the pancreatic protein PPARγ [33]. Neier et al. observed that the plasma levels of the adipokine MCP-1 decreased with increasing body fat in 10-month-old female offspring prenatally exposed to DBP. On the other hand, 8-month-old males showed a decrease in blood glucose at 120 min after glucose administration during oral glucose tolerance tests [32].

In 2020, Li et al., in agreement with previous studies, also found that offspring prenatally exposed to DBP showed an increase in fasting blood glucose levels and fasting serum insulin and leptin levels, showing that exposure to DBP leads to increased insulin resistance. This work also showed other parameters in common with previous studies, namely, an increase in serum and liver triglyceride levels and total cholesterol; however, it also found an increase in free fatty acids, meaning that the exposed offspring not only developed obesity but also dyslipidemia. Offspring that were prenatally exposed exhibited an increase in fat mass, as well as epididymal white adipose tissue (eWAT) and inguinal white adipose tissue (iWAT), at 21 weeks of age. In addition, they found an increase in the volume of adipocytes in the epididymal white adipose tissue, observing an increase in the lipids stored in the adipocytes. However, the rats’ activity level decreased slightly, as did their body temperature. Moreover, genomic assays were performed to analyze the expressions of several proteins related with brown adipocytes, thermogenesis, cell death and endoplasmic reticulum stress. The results show that exposure to DBP led to a decrease in the mRNA expression levels of UCP-1, Pgc-1α, Prdm16 and Cidea, and an increase in Bip and Chop proteins. Overall, the authors concluded that in utero exposure to DBP can increase stress in the endoplasmic reticulum, which inhibits the expression of UCP1, decreasing the energy consumption of brown adipose tissue, affecting the metabolism of lipids and sugars and leading to obesity in the offspring [31].

Two years later, in 2022, Zhou et al. observed an increase in visceral fat and a decrease in serum triglyceride levels in female offspring from PND7 to adulthood (PND90), in contrast to previous studies. This inconsistent result may be due to the use of a different species of rat. The offspring prenatally exposed to the highest concentration of DBP (132 mg/kg/day) showed a significant decrease in the expression of the G-protein-coupled estrogen receptor protein (GRP30) and an increase in the expression of protein kinase B (AKT), with no differences for the other signaling pathway proteins (PI3K, p-AKT, pAKT/AKT, PTEN, GLUT4, InsR, IRS). Despite there being no differences in the protein expression levels of the PTEN/PI3K/Akt signaling pathway in the muscle of the adult female F1 generation, the authors concluded that maternal perinatal exposure to DBP can induce visceral obesity in the offspring [36].

Overall, these studies show that prenatal exposure to DBP promotes the development of obesity as it increases the offsprings’ body weight [31,32,33,34,36]. In addition, exposure to this phthalate also caused an increase in the concentration of plasma cholesterol, triglycerides, free fatty acids and non-HDL cholesterol [31,33,35], showing that the exposed offspring developed dyslipidemia [31,35]. However, contrasting with the results obtained by the other studies, Zhou et al. observed a decrease in triglycerides in the exposed female offspring [36]. Furthermore, Negrin et al. found that the ingestion of corn oil, a commonly used vehicle, also increased serum levels of non-HDL cholesterol, and may thus potentiate the effects of DBP [35].

GDM occurs in pregnant women without previous diabetes who have been diagnosed with hyperglycemia, especially in the second or third trimester of pregnancy. The decreased ability of β-cells to proliferate can contribute to the development of this disease. The signal transducer and activator of transcription 1 protein (STAT 1) binds to the FoxM1 promoter (Forkhead box M1 protein) and contributes to diabetes by increasing the expression of genes involved in cell proliferation, fibrosis, inflammation and oxidative stress [41]. In this sense, Chen et al. used pregnant female Sprague-Dawley rats exposed only to 750 mg/kg/day of DBP or together with streptozotocin. This toxin specifically destroys the β-cells of the pancreatic islets of Langerhans and helps in the investigation of diabetes mellitus in animal models. DBP + streptozotocin-exposed offspring showed an increase in blood glucose and a decrease in insulin levels. In addition, FoxM1 expression levels were decreased due to it being negatively regulated by the phosphorylation of STAT1 (pSTAT1), which showed high expression levels. Thus, the authors concluded that the abnormal regulation of pSTAT1 and FoxM1 resulting from exposure to DBP is fundamental to the development of GDM [41].

Overall, DBP exposure caused dissimilar results, with some studies showing a decrease in insulin levels, while others showed an increase in serum insulin and leptin levels, as well as fasting plasma glucose concentration. Despite being contradictory, the results seem to show that DBP exposure leads to increased insulin resistance.

### 3.3. Renal System

The effects of DBP exposure on the renal system have been extensively studied in rats over the last 10 years. The male external genital organs originate from the genital tubercle through the cluster of mesenchymal cells in the cloacal membrane. Hypospadias, a congenital abnormality characterized by the opening of the urethra outside the tip of the penis, results from the inadequate fusion of the urethral folds and the arrest of growth of the urethral plate into the genital tubercle, and therefore apoptosis and autophagy are important for its normal development [42]. Autophagy is a protective mechanism enacted in response to different cellular injuries, such as hypoxia and DNA damage [43]. Some regulators of autophagy, such as mammalian target of rapamycin (mTOR), can inhibit autophagy through the Phosphatidyl-Inositol-3-Kinase (PI3K)/protein kinase B (Akt) signaling pathway, while Beclin 1 promotes it [44]. The activation of the PERK protein (Protein kinase R-like ER kinase) can inhibit protein synthesis through the phosphorylation of the eukaryotic translation initiation factor 2α (eIF2α) subunit in response to endoplasmic reticulum stress, regulating both apoptosis and autophagy [45]. Furthermore, long non-coding RNAs (lncRNAs) play a vital role in regulating autophagy and various reproductive processes [46]. Thus, the articles were divided according to the species analyzed, with particular emphasis on two primary areas of focus: hypospadias and renal fibrosis.

In this sense, Li et al. investigated the effect of prenatal exposure to DBP on the renal system using female Wistar rats exposed to 100, 300 or 900 mg/kg/day of DBP from E12.5 to E20.5 [47], while Zhu et al. and Sun et al. used pregnant female Sprague-Dawley rats exposed to 850 mg/Kg/day of DBP, but with different exposure periods, from GD11 to GD15 [48] and from GD12 to GD18 [49]. Using the same species, several researchers investigated the induction of hypospadias in pregnant female Sprague-Dawley rats exposed to 750 mg/kg/day of DBP from GD14 to GD18 [50,51,52,53,54]. However, Li et al. and Feng et al. changed the exposure period to GD13-GD18, with the latter also increasing DBP to 800 mg/kg/day [51,53]. Furthermore, Zhu et al. and Zhao et al. carried out consecutive complementary studies to investigate the impact of maternal exposure to DBP on the offspring’s renal system [43,55,56,57,58], exposing pregnant female Sprague-Dawley rats to 750 or 850 mg/Kg/day of DBP from GD14 to GD18 [43,55,57,58].

Regarding the results observed by Li et al., rats exposed to 900 mg of DBP showed a higher incidence of hypospadias, often accompanied by cryptorchidism. However, the subsequent administration of exogenous testosterone regulated the levels of this hormone and reduced the incidence of hypospadias in exposed rats [47]. In the research by Zhu et al., male offspring from rats exposed to DBP showed a 10.9% incidence of anorectal malformations combined with hypospadias, in addition to a diminished organ/BW ratio of the kidneys, lungs, heart and liver. This progeny exhibited a reduced expression of the androgen receptor (AR), which consequently decreased the expression of androgen-related genes, such as sonic hedgehog (Shh) and fibroblast growth factor 10 (Fgf10). This resulted in abnormal expressions of downstream factors (Gli2, Gli3, Bmp4, Wnt5a, Hoxa13, Hoxd13, Fgfr2) in the terminal rectum and genital tubercle [48].

Most studies observed that, after exposure to DBP, male offspring had a probability of hypospadias of between 42% and 49% [43,47,50,51,52,53], a decrease in the anogenital distance and the volume of the genital tubercle in the group exposed to DBP [51,52], and a more pronounced decrease in the size of the testes of the rats with hypospadias than those without deformity [50]. In male rats with hypospadias, the urethra opened ventrally along the axis of the genital tubercle, and the ventral tissue of the urethral plate was discontinuous. Also, in the testicles of non-deformed rats, some vacuoles were observed in the seminiferous tubules, and there was a decrease in lumen space. The testicles of rats with hypospadias showed irregular seminiferous tubules, more vacuoles, the absence of lumen and the hyperplasia of Leydig cells. Thus, DBP exposure can induce severe testicular dysplasia in rats with hypospadias, but not in non-deformed rats [50]. Jiang et al. and Li et al. found a more pronounced decrease in testosterone levels in rats with hypospadias [50,51]. There were also decreases in the expressions of the enzymes Cyp11a1, Hsd3b, Scarb1 and Star, and the expressions of AR and Srd5a2 were decreased only in the testes of rats with hypospadias [50]. Considering that Shh is essential for the formation of the genital tubercle and regulates the Bmp4, Fgf8 and Fgf10 androgen-related genes, their expressions were analyzed. The results show decreased expressions of Shh, Bmp4, Fgf8, Fgf10 and Fgfr2 in the genital tubercle in male rats with hypospadias. Jiang et al. concluded that relatively normal levels of testosterone-AR-Srd5a2 may contribute to resistance to DBP toxicity, preventing the development of hypospadias [50].

Different studies found a decrease in apoptosis that contributed to the development of hypospadias [51,52]. Furthermore, an increase in autophagy in the genital tubercle was also found, as the expressions of LC3-II and LC3B and the ratio LC3-II/LC3- I were increased, and the expressions of p62 and Beclin-1 were decreased [43,51,52,57,58]. In the genital tubercle of rats with hypospadias, the expressions of Akt, mTOR and S6 were reduced [51], while p-PERK, p-eIF2α and ATF4 proteins’ expressions increased [52]. The authors concluded that the increased autophagy in the genital tubercles of fetuses exposed to DBP in utero can be due to the inhibition of the PI3K/AKT/mTOR pathway [51] and the activation of the PERK-eIF2α pathway, which can also inhibit apoptosis, promoting the development of DBP-induced hypospadias [52]. In the study by Zhu et al., the results show that exposure to DBP increased the expression of IP3R, increasing intracellular calcium levels in urethral epithelial cells, given that IP3R is a calcium release channel in the endoplasmic reticulum. Besides this, E-cadherin, a calcium-dependent cell adhesion protein, showed elevated levels in the DBP-exposed group. In the genital tubercle of males with hypospadias, epithelial markers (E-cadherin, β-Catenin) were increased, while mesenchymal markers (Snail, N-cadherin) were decreased. Thus, exposure to DBP can influence the development of the genital tubercle by increasing oxidative stress, increasing cellular calcium concentration and inhibiting the epithelial–mesenchymal transition of urethral epithelial cells, contributing to the development of hypospadias [56]. Opposite to these results, other authors found a decrease in epithelial markers (E-cadherin) and an increase in mesenchymal markers (N-cadherin, VIM, β-catenin and Snail) in genital tubercles, suggesting that exposure to DBP promoted epithelial–mesenchymal transition in urothelial tissue [43,54,57]. Furthermore, Hua et al. observed that exposure to DBP increased the concentration of NAP-2 (cytokine potentially involved in the regulation of epithelial–mesenchymal transition) in the vascular endothelium and blood of pregnant mothers and their offspring. Thus, maternal exposure to DBP may lead to the excessive production of NAP-2, excessively activating the epithelial–mesenchymal transition of urothelial cells in genital tubercles and contributing to the development of hypospadias in male offspring [54]. The long non-coding (lnc) RNA NONRATT008453.2 regulates cellular autophagy by influencing the expressions of Atg5, Atg7, Beclin1 and LC3A/B proteins, and Feng et al. showed it to be the most highly expressed lncRNA in the group exposed to DBP. The authors also showed an increase in its expression in the fibroblasts of the genital tubercles of male offspring, suppressing autophagy, concluding that lncRNA NONRATT008453.2 can affect autophagy in genital tubercle fibroblasts in male rats with DBP-induced hypospadias [53].

Zhu et al., Sun et al. and Zhao et al. observed that exposure to DBP resulted in decreased body weight and kidney size in rats, as well as tubular damage, increased interstitial space, and thickened tubular basement membrane [49,55,57]. The expressions of genes involved in kidney development (Foxd1, Wnt11, Pax2, Gdnf) were reduced, while the expressions of genes related to kidney structure (Bmp4, Cdh11, Ywhab, Calm1) were increased, suggesting that exposure to DBP may lead to incomplete kidney development. There was also an increase in the expression of matrix components such as alpha smooth muscle actin (α-SMA), fibronectin, and TGF-β, indicating that maternal exposure to DBP can induce renal dysplasia at PND1 and renal fibrosis in adulthood [49,55]. On PND70, Sun et al. observed a decrease in serum testosterone concentration, AR expression, and Fgf10 and Fgfr2 proteins in male offspring with renal fibrosis [49]. As the transcription of Fgf10 and Fgfr2 depends on AR signaling, exposure to DBP disrupted the Fgf10/Fgfr2 signaling pathway in the kidneys, this being a crucial factor in the development of renal fibrosis [49]. Similarly, in the following year, Zhao et al. also observed a decrease in serum testosterone and AR expression in rats with hypospadias, suggesting that DBP promotes the development of hypospadias through abnormal autophagy and epithelial–mesenchymal transition [43]. In the study performed by Zhao et al., maternal exposure to DBP was suggested to be involved in the development of renal fibrosis, considering that the expression of TGF-β1, which is important in the regulation of renal fibrosis and mesenchymal–epithelial transition (MET), was increased [57]. In 2020, Zhao et al. investigated the hedgehog signaling pathway, essential for normal embryonic development, and found that the expression of the negative regulator HhIP was elevated in the group exposed to DBP, while the expressions of Gli1 and Ptch1, transcriptional targets of hedgehog signaling pathway, were decreased. Thus, exposure to DBP can inhibit the hedgehog signaling pathway, resulting in poor kidney development [58].

Overall, the studies analyzed show that exposure to DBP during pregnancy has a significant impact on the development of the male renal system, especially in the induction of hypospadias in rats. Although there is still no complete agreement on the underlying mechanisms, they seem to involve a complex interaction of cellular processes, such as autophagy and apoptosis, and the regulation of epithelial–mesenchymal transition.

### 3.4. Digestive System

Anorectal malformations are congenital diseases that result from abnormal anorectal development, such as the obstruction of the anal opening, being more common in men [59]. Anorectal development is mediated by complex signaling pathways including Shh and fibroblast growth factor 10 (Fgf10), as well as downstream factors such as Gli2, Gli3, bone morphogenetic protein 4 (Bmp4), Wnt5a, Hoxa13, Hoxd13 and fibroblast growth factor receptor 2 (Fgfr2). Shh and Fgf10 are mediated by androgens during embryonic development. The Wnt5a protein, expressed mainly in the colon and rectum during embryonic development, is essential for the normal growth of several structures, including the gastrointestinal tract [60].

Regarding anorectal development and its malformations after exposure to DBP, three studies were carried out. Thus, Li et al., Jiang et al. and Liu et al. exposed pregnant female Sprague-Dawley rats to 850 mg/kg/day of DBP from GD12 to GD18 [5,61,62]. Jiang et al. and Liu et al. found a 39.5% incidence of anorectal malformations in male offspring prenatally exposed to DBP, including anal atresia and accumulated meconium [5,61,62]. None of the studies examined the anal structure or the transition zone between the epithelial cells of the rectal gland and the perianal squamous cells. Furthermore, the blind side of the terminal rectum was covered with intestinal epithelium in the exposed rats, while controls showed normal anal openings and transition zones [5,61,62]. Li et al. observed that maternal toxicity emerged after three days of exposure to DBP, with a decrease in maternal body weight between GD15 and GD18. Besides this, when comparing the effects of DBP exposure between the groups with and without anorectal malformations, it was found that the expression of Wnt5a decreased markedly in the group with anorectal malformations, suggesting that DBP may induce anorectal maldevelopment by disturbing the signaling pathway mediated by Wnt5a [62]. In the studies by Jiang et al. and Liu et al., there was a decrease in body weight and anogenital distance in rats exposed to DBP [5,61], and also a reduction in the size of the heart, lungs, liver, spleen and kidneys [5]. Jiang et al. also noted decreased androgen levels in the anorectal and renal tissues of male rats with anorectal malformations, indicating that exposure to DBP may disrupt AR signaling, leading to anorectal malformations and renal dysplasia. A reduced expression of Fgf10/Fgfr2 was observed in the terminal rectum, kidney, spleen, liver and heart of male rats with anorectal malformations, with no alterations in AR expression in the spleen, liver and heart. According to these results, it is suggested that the AR and the Fgf10/Fgfr2 signaling pathways could be involved in the development of anorectal malformations in male rats exposed to DBP in utero [61]. Liu et al. also observed a decrease in serum testosterone and mRNA levels of the Shh, Gli2, Gli3, Bmp4, Wnt5a, Hoxa13, Hoxd13, Fgf10, Fgfr2 and AR genes in male offspring with anorectal malformations [5]. Thus, exposure to DBP suppresses androgen production and AR expression, interfering with the activation of androgen-related genes, leading to the abnormal expression of downstream factors, and contributing to the development of anorectal malformations [5].

In summary, studies on DBP exposure in animal models have demonstrated that it can induce significant anorectal malformations in male offspring. Exposure to DBP interferes with androgen-mediated signaling pathways such as Shh and Fgf10, reducing the expression of genes essential for anorectal development, and resulting in abnormalities in the development of the digestive system.

**Table 1:** Summary of the effects of DBP derived from the in vivo studies on nervous system, metabolism, renal system and digestive system.

System	Reference	Animal	DBP Dose	Solvent	Route of Exposure	Duration of Exposure	Main Conclusions
Nervous	Li et al., 2014 [26]	Sprague-Dawley rats	500 mg/Kg/day	Corn oil	Intragastically	From GD6–PND21	**1.** Immature offspring showed increased expressions of AROM and decreased expressions of ER-β, BDNF and CREB in the hippocampus, which may contribute to DBP-induced neurotoxicity **2.** No significant results obtained in mature offspring, which could mean that immature animals can recover over time
	Wang et al., 2019 [21]	White Leghorn chick eggs	0.1–1000 µM		Injected directly into the egg yolk		**1**. DBP led to the development of neural tube defects, decreased body weight, and increased malformations in the embryos **2**. Embryos treated with 1 µM DBP showed poor development of the vascular chorioallantoic membrane **3**. Exposure to DBP increased oxidative stress levels and apoptosis **4**. A high concentration of choline protected embryos from DBP-induced embryonic malformations and inhibited the development of oxidative stress
	Lee et al., 2020 [29]	C57BL/6 mice	50 and 100 mg/kg/day	Corn oil	Oral	From GD13–PND15 (lactation period)	**1**. Decreased scores in motor development and memory tests **2**. Delayed latency period in females and poor maternal behavior **3**. Positive regulation of genes associated with the dopamine system, which could cause hyperactivity in the offspring and a delay in neurological development
	Hunter et al., 2021 [27]	Sprague Dawley rats	500 mg/kg	Corn oil	Oral every two days	From GD14.5–PND6	**1**. Reduced expression of the Avp gene and increased expression of the Gnrh1 gene **2**. Increased latency period in male offspring **3**. Female offspring remained sexually receptive
	Xia et al., 2022 [28]	BALB/c mice	50, 250 and 500 mg/kg/day	Corn oil	Intragastically	From GD12.5–GD21.5	**1**. Increased activation of astrocytes through activation of the JAK2/STAT3 signaling pathway, which affected the morphology of the dendritic spines and abnormally increased the synaptic formation of neurons in the hippocampus, leading to a decrease in the autonomous and exploratory behavior of the offspring
	Radha and Basha 2022 [30]	Wistar rats	500 mg/kg/day	Olive oil	Oral	From GD6–GD21 and during lactation	**1**. Decreased SOD and CAT (antioxidant enzymes) in the neuronal tissue leading to the development of oxidative damage and DNA damage through lipid peroxidation, which was also elevated in the F3 generation rats in all areas of the neuronal tissue studied **2**. Decreased contents of nucleic acids in all areas of the brain and increased damage to DNA chains, with a higher incidence in the F3 generation
	Wakui et al., 2013 [38]	Sprague-Dawley rats	100 mg/kg/day	Corn oil	Intragastically	From GD12–GD21	**1.** The body weight of the male offspring was not affected by DBP exposure
	Wakui et al., 2014 [37]	Sprague-Dawley rats	100 mg/kg/day	Corn oil	Intragastically	From GD12–GD21	**1.** The body weight of the male offspring was not affected by DBP exposure
	Chen et al., 2017 [39]	Sprague-Dawley rats	100, 500 mg/kg/day	Corn oil	Oral	From GD12–GD21	**1.** The body weight of the male offspring was not affected by DBP exposure
	Okayama et al., 2017 [40]	Sprague-Dawley rats	100 mg/kg/day	Corn oil	Oral	From GD12–GD21	**1.** The body weight of the male offspring was not affected by DBP exposure
	Christante et al., 2018 [34]	Mongolian gerbil	100 mg/kg/day	Mineral oil	Oral	From GD8–GD23	**1.** Increased body weight of male offspring
	Negrin et al., 2018 [35]	Mongolian gerbil	100 mg/kg/day	Corn oil	Oral	From GD8–GD23	**1.** Exposure to DBP led to dyslipidemia with increased serum levels of triglycerides, total cholesterol and non-HDL cholesterol **2.** Ingestion of corn oil also increased serum levels of non-HDL cholesterol and may thus potentiate the effects of DBP
Metabolism	Venturelli et al., 2019 [33]	Wistar rats	5, 50 and 500 mg/kg/day	Canola oil	Oral	From GD13–GD21	**1**. Increased offspring’s body weight during puberty and lactation **2**. Adult males in the group exposed to 5 mg of DBP showed an increase in plasma cholesterol concentration **3**. Both males and females in the group exposed to 500 mg of DBP showed an increase in fasting plasma glucose concentration, and females showed an increase in triglyceride concentration
	Neier et al., 2019 [32]	a/a mice (longitudinal model)	25 mg/Kg/day	Corn oil	Oral	From 2 weeks before mating until PND21	**1**. Increased body weight of female offspring **2**. Plasma levels of MCP-1 decreased with increasing body fat in 10-month-old female offspring **3**. Decreased blood glucose at 120 min after glucose administration during oral glucose tolerance in 8-month-old males
	Li et al., 2020 [31]	SPF C57BL/6J mice	5 mg/kg/day	Corn oil	Oral	From GD12–PND7	**1**. Increased body weight of offspring **2**. Increased fat mass, epididymal and inguinal white adipose tissue **3**. Increased stored lipids in fat cells **4**. Increased fasting blood glucose and insulin levels **5**. Development of obesity and dyslipidemia **6.** DBP exposure may increase endoplasmic reticulum stress, which inhibits UCP1 expression, decreasing brown adipose tissue energy consumption and affecting lipid and sugar metabolism, consequently leading to obesity in the offspring
	Chen et al., 2020 [41]	Sprague-Dawley rats	750 mg/Kg/day	Peanut oil	Gastrically	From GD1–GD3	**1**. Offspring prenatally exposed to DBP + streptozotocin showed an increase in blood glucose concentration and a decrease in insulin levels **2**. FoxM1 expression levels were decreased due to its negative regulation by the phosphorylation of STAT1 (pSTAT1), which showed high expression levels **3**. The abnormal regulation of pSTAT1 and FoxM1 resulting from exposure to DBP is fundamental to the development of gestational diabetes mellitus
	Zhou et al., 2022 [36]	Sprague-Dawley rats	33, 66 and 132 mg/kg/day	Corn oil	Intraperitoneally	From GD7–PND21	**1**. No significant differences in the protein levels of the PTEN/PI3K/Akt signaling pathway in the muscle tissue of the adult female offspring of the F1 generation **2**. Maternal perinatal exposure to DBP can induce visceral obesity in the offspring
Renal System	Li et al., 2015 [47]	Wistar rats	100, 300 and 900 mg/kg/day	Corn oil	Intragastrically	From E12.5–E20.5	**1.** Rats exposed to 900 mg of DBP had increased incidence of hypospadias, often accompanied by cryptorchidism **2**. The administration of exogenous testosterone regulated testosterone levels and reduced the incidence of hypospadias in exposed rats
	Jiang et al., 2016 [50]	Sprague-Dawley rats	750 mg/kg/day	Corn oil	Gastric intubation	From GD14–GD18	**1**. Relatively normal levels of testosterone-AR-Srd5a2 may contribute to resistance to DBP toxicity, preventing the development of hypospadias
	Zhu et al., 2016 [48]	Sprague-Dawley rats	850 mg/kg/day	Corn oil	Gastric intubation	From GD11–GD15	**1.** Maternal exposure to DBP led to a 10.9% incidence of anorectal malformations combined with hypospadias in the male offspring **2**. Reduced expression of AR, which decreased the expression of androgen-related genes (Shh and Fgf10), which resulted in abnormal expression of downstream factors (Gli2, Gli3, Bmp4, Wnt5a, Hoxa13, Hoxd13, Fgfr2) in the terminal rectum and genital tubercle
	Li et al., 2017 [51]	Sprague-Dawley rats	750 mg/kg/day	Corn oil	Oral	From GD13–GD18	**1**. Exposed male offspring with a 43.64% incidence of hypospadias **2**. Decreased anogenital distance and genital tubercle volume **3**. Decreased testosterone in rats with hypospadias **4**. Decreased apoptosis and increased autophagy **5**. Decreased expression of Akt, mTOR and S6 in the genital tubercle of rats with hypospadias, and inhibition of the PI3K/AKT/mTOR pathway increased autophagy in the genital tubercles of fetuses exposed to DBP
	Zhu et al., 2017 [55]	Sprague-Dawley rats	850 mg/kg/day		Oral perfusion	From GD14–GD18	**1**. Reduced expression of genes involved in kidney development (Foxd1, Wnt11, Pax2, Gdnf), while increased expression of genes related to kidney structure (Bmp4, Cdh11, Ywhab, Calm1), suggesting that exposure to DBP can lead to incomplete kidney development **2**. Increased expression of α-SMA proteins, fibronectin and TGF- β **3**. Maternal exposure to DBP can induce renal dysplasia in PND1 and renal fibrosis in adulthood
	Zhao et al., 2018 [52]	Sprague-Dawley rats	750 mg/kg/day	Corn oil	Gastric intubation	From GD14–GD18	**1**. Exposure to DBP led to a 43.48% incidence of hypospadias in male rats **2**. Decreased anogenital distance and genital tubercle volume **3**. Decreased cell apoptosis and increased cell autophagy in the genital tubercle of rats with hypospadias **4**. Increased expression of p-PERK, p-eIF2α and ATF4 proteins in the genital tubercle of male rats with hypospadias, concluding that activation of the PERK-eIF2α pathway may increase cellular autophagy and inhibit apoptosis, promoting the development of hypospadias induced by DBP
	Zhao et al., 2018 [43]	Sprague-Dawley rats	750 mg/kg/day	Corn oil	Gastric intubation	From GD14–GD18	**1**. Exposure to DBP led to a 42.3% incidence of hypospadias in male rats **2**. Increased autophagy of the genital tubercle **3**. DBP promotes the development of hypospadias through abnormal autophagy and epithelial–mesenchymal transition resulting from the stress caused by DBP
	Sun et al., 2018 [49]	Sprague-Dawley rats	850 mg/kg/day	Corn oil	Gastric intubation	From GD12–GD18	**1**. Decreased body weight and kidney size in male offspring with renal fibrosis **2**. Increased expression of matrix components (α-SMA and TGF-β) **3**. Decreased serum testosterone concentration, AR expression and Fgf10 and Fgfr2 proteins in male offspring with renal fibrosis
	Zhao et al., 2019 [57]	Sprague-Dawley rats	850 mg/kg/day	Corn oil	Oral	From GD14–GD18	**1**. Decreased size of the fibrotic kidneys of the offspring with thickening of the tubular basement membrane and tubular damage **2**. Increased expression of TGF-β1 **3**. Maternal exposure to DBP promotes Snail1-mediated epithelial–mesenchymal transition through up-regulation of TGF-β1, leading to the development of renal fibrosis
	Zhu et al., 2020 [56]	Sprague-Dawley rats	750 mg/kg/day	Corn oil	Gastric intubation	From GD14–GD18	**1**. DBP exposure increased IP3R expression, raising intracellular calcium levels in urethral epithelial cells **2**. Increased epithelial markers (E-cadherin, β-Catenin) in the genital tubercle of males with hypospadias, and decreased mesenchymal markers (Snail, N-cadherin), inhibiting the epithelial–mesenchymal transition and contributing to the development of hypospadias
	Zhao et al., 2020 [58]	Sprague-Dawley rats	850 mg/kg/day	Corn oil	Gastric intubation	From GD14–GD18	**1**. Increased expression of the negative regulator HhIP in the DBP-exposed group, and decreased expression of Gli1 and Ptch1 **2**. DBP exposure inhibited the hedgehog signaling pathway, resulting in poor kidney development
	Feng et al., 2021 [53]		800 mg/kg/day	Corn oil	Oral	From GD13–GD18	**1.** The lncRNA NONRATT008453.2 affected autophagy in genital tubercle fibroblasts in male rats with DBP-induced hypospadias **2**. Overexpression of lncRNA NONRATT008453.2 decreased fibroblast proliferation and suppressed autophagy in these cells
	Hua et al., 2023 [54]	Sprague-Dawley rats	750 mg/kg/day	Corn oil	Oral	From GD14–GD18	**1**. Increased development of hypospadias in offspring **2**. Mice with hypospadias showed decreased epithelial markers (E-cadherin) and increased mesenchymal markers (N-cadherin and VIM) in the genital tubercles **3.** Increased concentration of NAP-2 **4**. Maternal exposure to DBP can lead to excessive production of NAP-2, excessively activating the epithelial–mesenchymal transition of urothelial cells in the genital tubercles and contributing to the development of hypospadias in the male offspring
Digestive System	Li et al., 2014 [62]	Sprague-Dawley rats	850 mg/kg/day	Corn oil	Gastric intubation	From GD12–GD18	**1**. Decrease in maternal body weight from GD15 to GD18 **2**. In PND1, anal atresia and accumulated meconium in the offspring with anorectal malformations. The blind side of the terminal rectum was covered in intestinal epithelium **3**. Decreased Wnt5a expression in the DBP-exposed group with anorectal malformations
	Jiang et al., 2015 [61]	Sprague-Dawley rats	850 mg/kg/day	Corn oil	Gastric intubation	From GD12–GD18	**1**. In PND1, 39.5% incidence of anorectal malformations in male offspring prenatally exposed to DBP **2**. Decreased body weight and anogenital distance, anal atresia and accumulated meconium **3**. In PND1, decreased concentration of serum testosterone **4**. Decreased expression of AR in the terminal rectum and kidney **5**. Decreased expression of Fgf10 and Fgrf2 in the terminal rectum, kidney, spleen, liver and heart **6**. The blind side of the terminal rectum was covered with intestinal epithelium in the exposed rats
	Liu et al., 2016 [5]	Sprague-Dawley rats	850 mg/kg/day	Corn oil	Gastric intubation	From GD12–GD18	**1**. In PND1, 39.5% incidence of anorectal malformations in male offspring with anal atresia and accumulated meconium **2**. Decreased body weight and anogenital distance **3**. In male rats with anorectal malformations, the blind side of the terminal rectum was covered in intestinal epithelium **4**. DBP exposure suppresses androgen production and AR expression, interfering with the activation of androgen-related genes, such as Shh and Fgf10, and leading to the abnormal expression of downstream factors, contributing to the development of anorectal malformations

### 3.5. Male Reproductive System

Spermatogenesis takes place in the seminiferous tubules of the testicles of rodents and humans, which are composed of peritubular myoid cells, Sertoli cells and male germ cells [63]. Sertoli cells support testicular somatic cells and Leydig cells, which are found in the interstitial space between the seminiferous tubules and produce testosterone through steroidogenesis in the mitochondria and smooth endoplasmic reticulum [63]. These cells also express ERα, ERβ and AR [37,64]. There are two generations of Leydig cells, premature and adult, which are essential for sexual maturation, male mating behavior and fertility [37]. In the testicles of rats, the fetal Leydig cells begin to involute and practically disappear on the PND21. On PND14, spindle-shaped progenitor Leydig cells appear and differentiate into immature Leydig cells on PND28, which in turn differentiate into adult Leydig cells on PND49. These cells begin to produce their maximum testosterone at PND56 [65]. The synthesis of androgens in Leydig cells begins with the conversion of cholesterol by the P450 side chain cleavage enzyme (CYP11A1, encoded by the Cyp11a1 gene). The subsequent enzymatic reactions leading up to the formation of testosterone are catalyzed by the enzymes 3β-hydroxysteroid dehydrogenase 1 (3β-HSD1, encoded by the Hsd3b1 gene), 17α-hydroxylase (CYP17A1, encoded by the Cyp17a1 gene) and 17β-hydroxysteroid dehydrogenase 3 (17β-HSD3, encoded by the Hsd17b3 gene). Androgen production in Leydig cells is regulated by luteinizing hormone (LH), which in turn is regulated by gonadotropin-releasing hormone (GnRH). The steroidogenic acute regulatory protein (StAR) is important for androgen synthesis, as it is responsible for transporting cholesterol to the inner membrane of the mitochondria [66].

Regarding the effect of DBP after prenatal exposure on the male reproductive system, there are only four studies on Leydig cells. The first authors to analyze this effect were Wakui and collaborators, who investigated the morphology of rats’ Leydig cells hyperplasia and changes in the hormone receptors (ERα, ERβ, AR) during puberty and adulthood after exposure to DBP [37,38]. Motohashi et al. [64] investigated the correlation between the morphological alteration of the smooth endoplasmic reticulum of Leydig cells and the expression of steroidogenesis enzymes after in utero exposure to DBP [64]. Chen et al. investigated whether prenatal exposure to DBP affected the involution of fetal Leydig cells during the neonatal period, and whether this event caused the delayed development of adult Leydig cells during puberty [39]. Briefly, these authors exposed pregnant female Sprague-Dawley rats to 100 mg/kg/day of DBP, and also to 500 mg/kg/day, from GD12 to GD21 [37,38,39,64]. The authors found that testicular weight after prenatal exposure to DBP decreased from 9 weeks of age [37,38,64]. Treatment with this phthalate also led to a reduction in the smooth endoplasmic reticulum of Leydig cells [38], with Motohashi et al. observing its disappearance from 17 weeks of age [64]. All the studies found a decrease in testosterone levels, which Wakui et al. attributed to the reduction in smooth endoplasmic reticulum and hyperplasia of Leydig cells [38]. Besides this, Wakui et al. and Motohashi et al. found an increase in the number of Leydig cells in the groups exposed to DBP [37,64], while Chen et al. found that, on PND7, both the group exposed to 100 mg/kg/day and that exposed to 500 mg/kg/day of DBP showed an increase in the number of fetal Leydig cells [39]. However, on PND14 and PND21, the group exposed to 100 mg of DBP showed similar levels to the control, while the group exposed to 500 mg of DBP showed an even greater increase in the number of fetal Leydig cells [39]. These authors also found a DBP dose-dependent reduction in cell size, without affecting the size of the nucleus [39]. However, Wakui et al. observed that the Leydig cells presented atypical nuclei, anomalous occlusion junctions and abundant microfilament components, and lacked microbodies [38].

The following year, this research group observed that between 9 and 17 weeks of age, the offspring prenatally exposed to DBP showed severe testicular degeneration and dysplastic hyperplasia of the Leydig cells [37]. In the DBP-exposed group, there was also a decrease in serum LH concentration between 5 and 7 weeks of age, followed by an increase between 9 and 17 weeks of age, in which an increase in the expression of both ERα protein and mRNA was also demonstrated, while ERβ and AR decreased. The elevated LH levels were justified as a compensatory reaction to the decrease in testosterone, and the authors concluded that prenatal exposure to DBP can cause Leydig cell hyperplasia, probably due to increased LH levels, low testosterone levels and elevated ERα expression in these cells [37]. In the study by Motohashi et al. after DBP in utero exposure, the low levels of testicular testosterone during the juvenile and peri-pubertal periods (5–17 weeks of age) were due to the downregulation of StAR and Cyp11a1, while during peri-puberty and adulthood (9–17 weeks of age), they were due to the downregulation of Hsd3b, Cyp17a1 and Hsd17b3, important enzymes associated with the smooth endoplasmic reticulum. However, it was not possible to determine whether the negative regulation of these enzymes is the cause for the decrease or disappearance of the smooth endoplasmic reticulum in this period [64]. Chen et al. found that DBP increased the number of large clusters of fetal Leydig cells, delaying their disappearance and increasing their number during PND14 to PND21, a critical period for the development of adult Leydig cells. At PND28 and PND56, a reduction in the number of adult Leydig cells was observed in the group exposed to 500 mg of DBP, suggesting a delay in their development. In this group, several Leydig cell-specific genes, such as Scarb1, StAR, Cyp11a1, Hsd3b1, Hsd11b1 and Hsd17b3, were negatively regulated from PND7 to PND56 [39]. The negative regulation of GnRH resulted in a decrease in LH expression from PND3 to PND56, which contrasts with the results obtained by Wakui et al. [37]. Chen et al. concluded that reduced LH could also contribute to delayed Leydig cell development [39]. Reduced testosterone levels were justified by the reduction in smooth endoplasmic reticulum and Leydig cells’ hyperplasia caused by increased LH levels, low testosterone levels and elevated ERα expression in these cells [37], while Motohashi et al. and Chen et al. found that several specific genes of the Leydig cells were negatively regulated, leading to a testosterone decrease [39,64].

As for the Sertoli cells, they surround the spermatogonia along the basal membrane of the testicular seminiferous tubules, which, in turn, are surrounded by the peritubular myoid cells [67]. The formation of the seminiferous tubules ends on embryonic day 14.5 (E14.5) in mice [68]. After the formation of the tubules, the fetal Leydig cells differentiate, starting to produce insulin-like factor 3 (INSL3) and testosterone, which, together with the anti-Müllerian hormone produced by the Sertoli cells, are responsible for the masculinization of the internal and external genital organs, during the “masculinization programming window (MPW)” that occurs between E15.5 and E18.5 [69], corresponding to a peak in circulating testosterone that occurs at E17.5 [70]. This period occurs between the 8th and 14th gestational weeks in humans [69].

The inflammatory mediator cyclooxygenase-2 (COX-2) can be regulated by nuclear factor-κB (NF-κB) and plays a role in regulating spermatogenesis. RANKL, a transmembrane ligand expressed in various cells, including Sertoli cells, regulates cell proliferation, differentiation and apoptosis [71]. In this regard, there are currently three studies on the effect of DBP exposure on Sertoli cells. In the first study, Okayama et al. studied the morphological changes in the Sertoli cells of male offspring, using pregnant female Sprague-Dawley rats exposed to 100 mg/kg/day of DBP from GD12 to GD21 [40]. Regarding the second study, the origin of ectopic Sertoli cells was investigated, while Xie et al. evaluated the possible association of the NF-κB/COX-2/RANKL signaling pathway with impaired spermatogenesis [68,71].

Okayama et al. found that at 14 and 17 weeks of age, male offspring prenatally exposed to DBP showed testicular atrophy characterized by smaller testes, seminiferous tubules with a reduced diameter and an increase in the number of Sertoli cells. The study concluded that prenatal exposure to DBP resulted in an increase in the number and proliferation of Sertoli cells from puberty to adulthood, with a decrease in testicular testosterone and an increase in serum follicle-stimulating hormone (FSH) [40]. Regarding the study conducted by Lara et al., these authors used pregnant female rats exposed to DBP (750 mg/kg/day) during the following periods: FW (full window), from E13.5 to E20.5; MPW, from E15.5 to E18.5; or LW (late window), from E19.5 to E20.5 [68]. The results show that E21.5 offspring prenatally exposed during MPW and FW showed an increase in ectopic germ cells and Sertoli cells in the interstitial space. However, in those exposed during MPW, where the treatment started after normal tubule formation, there was an abnormal aggregation of fetal Leydig cells at E17.5, showing the first signs of focal dysgenesis. In addition, the peritubular myoid cells showed reduced functionality, which led the authors to conclude that the increased number of germ cells migrating to the basal lamina in the animals exposed to DBP during MPW leads to the rupturing of the weakened basal lamina, explaining the increased number of germ cells and ectopic Sertoli cells in the dysgenic areas during MPW compared to FW, where the animals showed lower germ cell migration. The focal dysgenic areas in the fetal testes transform into focal areas with abnormal seminiferous tubules after the birth of the offspring [68]. Lastly, Xie et al., who exposed pregnant female Sprague-Dawley rats from GD14 to GD18 to 750 mg/kg/day of DBP, found an increase in the expression of RANKL mRNA and protein and a decrease in spermatogonia in the seminiferous tubules of the offspring [71]. In summary, these studies allow us to infer that after exposure to DBP, the expression of RANKL in Sertoli cells increased through the NF-κB/COX-2 signaling pathway, as verified in in vitro assays, resulting in the apoptosis of spermatogonia [71]. Besides this, Lara et al. concluded that the rupture of the basal lamina led to an increase in the number of germ cells and ectopic Sertoli cells in the interstitial space, which was also confirmed by Okayama et al. [40,68].

Testosterone is one of the main androgens that regulate the progression of spermatogenesis in mammals [72]. There are currently six studies analyzing concentrations of male hormones, particularly testosterone. Specifically, in 2014, Giribabu et al. exposed pregnant female Albino Wistar rats to 100 or 500 mg/kg/day of DBP on GD1, GD7 and GD14 [73]. Pike et al. examined the foreskins of rats, which only develop after GD18.5, to detect changes in the gene expression of the androgen signaling pathway. For this, pregnant female Sprague-Dawley rats were exposed to 100 or 500 mg/kg/day of DBP from GD16 to GD20 [70]. In the same year, Lourenco et al. analyzed the influence of different oils as vehicles on DBP-induced testicular toxicity, using pregnant female Wistar rats exposed to 500 mg/kg/day of DBP from GD13 to GD20 [74]. The following year, Li et al. exposed pregnant female Wistar rats to 100, 300 or 900 mg/kg/day of DBP, from E12.5 to E20.5, to investigate the effect of this phthalate on the male reproductive system [47]. Zhu et al. exposed pregnant female Sprague-Dawley rats to 850 mg of DBP/kg/day from GD11 to GD15 [48], and a few years later, Giribabu and Reddy 2017 investigated whether the intra-peritoneal administration of testosterone (4.16 mg/kg) on PND35, PND55 and PND75 could protect the Wistar rats from DBP-induced reproductive toxicity [75].

In the two studies carried out by Giribabu’s research group, it was found that exposure to DBP resulted in a decrease in the number of offspring and in the prenatal survival rate, effects that were reversed with testosterone supplementation. In addition, testosterone increased the weight of the testes and seminal vesicle, and improved sperm count, motility and viability, reducing the incidence of morphologically altered sperm [73,75]. In the testes, rats exposed to 100 mg of DBP showed disorganization of the seminiferous tubules and rupture of the epithelium, while those exposed to 500 mg of DBP had elongated tubules devoid of sperm [73]. After testosterone supplementation, there was partial recovery of testicular architecture [75]. Exposure to DBP decreased the activity of 3β-HSD1 and 17β-HSD3 enzymes and serum testosterone levels, while increasing FSH and LH levels in the testes of adult rats. Testosterone administration reversed these changes, increasing enzyme activities and testosterone levels and reducing FSH and LH due to negative feedback [73,75]. Furthermore, exposure to DBP increased lipid peroxidation (LPO) and reduced the activity of the antioxidant enzymes SOD, CAT and glutathione peroxidase (GPx) in the testes, effects that were also reversed by testosterone, demonstrating its ability to reduce oxidative stress [75]. These results show that prenatal exposure to DBP reduces reproductive performance in adulthood due to lower steroidogenesis and reduced sperm quality and quantity, which can lead to infertility. However, testosterone has antioxidant properties, and its supplementation antagonizes the reproductive toxicity caused by DBP [73,75].

In the same sense, Pike et al. observed that fetuses exposed in utero to DBP showed an increase in seminiferous tubules with multinucleated gonocytes. Unlike the previous study, only DBP 500 mg resulted in a significant decrease in testosterone levels in the testicles. This exposure also led to a decrease in anogenital distance detected on GD20 and PND5. Furthermore, 71% of offspring prenatally exposed to 500 mg of DBP experienced nipple retention. The Marcks, Pum1 and Slc7a1 genes, involved in the downstream effects of AR signaling, were identified at both GD20 and PND5 after exposure to 500 mg DBP, with Marcks and Pum1 showing decreased expression. Thus, these authors concluded that Marcks, Pum1, Penk, and Nupr1 may serve as postnatal markers of prenatal DBP exposure [70]. Still, in 2014, Lourenco et al. found similar results when comparing corn, canola and fish oils as DBP vehicles, that is, exposure to DBP reduced anogenital distance and testicular testosterone levels in offspring, regardless of the vehicle used [74]. Although all groups, including controls, presented multinucleated gonocytes in the seminiferous tubules, these were increased in the groups exposed to DBP, along with the diameter of the tubules, as observed by Pike et al. [70]. Furthermore, exposure to DBP led to an increase in large clusters of Leydig cells and a decrease in small clusters in the offspring. Thus, this study led to the conclusion that the different vehicles did not affect fetal testicular toxicity induced by prenatal exposure to DBP and, consequently, did not contribute to the variability of responses [74]. Regarding the study performed by Li et al., it was found that the group exposed to 300 mg of DBP showed a reduction in testicular weight, in the number of Leydig cells and in the levels of testicular testosterone on E17.5, although the values were practically normalized until E19.5. Additionally, the authors observed a decrease in anogenital distance, but no cryptorchidism was observed. On the other hand, in the group exposed to 900 mg of DBP, there was a decrease in anogenital distance and testicular weight, with a 17.4% incidence of cryptorchidism. There was also a suppression of testosterone synthesis after E15.5, in addition to aggregation and a decrease in the number of Leydig cells. Thus, the authors concluded that exposure to lower concentrations of DBP may cause a reversible delay in testicular development, while higher concentrations can induce irreversible testicular damage.

Furthermore, the authors also administered exogenous testosterone to the animals, and observed that testosterone levels were normalized and there was an increase in anogenital distance in embryos exposed to 300 and 900 mg of DBP [47]. These results are consistent with the study carried out by Giribabu and Reddy (2017) [75]. In 2016, the study carried out by Zhu et al. observed similar results, with a decrease in anogenital distance, serum testosterone concentration and testicular size, which showed a disorganized structure of the seminiferous tubules. Similarly to Wakui et al. [37], this study also found Leydig cell hyperplasia and an abnormally shaped lumen. The expressions of Shh and Fgf10 genes, related to the androgen signaling pathway in the testis, were reduced. The authors concluded that testicular atrophy resulted in a decrease in the reproductive capacity of male offspring, contributing to a decrease in serum testosterone [48].

Regarding the appearance of multinucleated germ cells (MNGs) and the abnormal aggregation of gonocytes in the center of the seminiferous tubules, a study was carried out in 2015 using 20 female Sprague-Dawley rats treated with 500 mg/kg/day of DBP from GD17, and 35 female Sprague-Dawley rats treated from GD18. DBP doses were administered at 0 h and 24 h, and the animals were euthanized at 0, 2, 4, 6, 24, or 48 h after the initial dose. Initially, gonocytes undergo mitotic expansion during gestation, and, on the GD18, they enter a quiescent state before differentiating into spermatogonia. Germ cell apoptosis is common during the mitotic phase but rare after the quiescent state, except for a wave of spermatogonial apoptosis that occurs after birth. Thus, the authors observed that exposure to DBP altered the morphology of the seminiferous tubules in the first 24 h after initial exposure, only when the treatment began on GD18. In this group, there was an increase in the diameter of the seminiferous tubules and in the rate of MNGs after 24–48 h of exposure to DBP. In contrast, in utero exposure to DBP starting on GD17 did not significantly affect cell death in the seminiferous tubules, while exposure starting on GD18 resulted in increased cell death at 48 h. Spade et al. concluded that exposure to DBP alters the morphology of the fetal seminiferous tubules without having significant effects on testicular cell proliferation or cell death. Furthermore, the researchers discovered that MNGs are formed through a mechanism independent of germ cell proliferation [76].

Prostate morphogenesis depends on androgens and results from the interaction between the epithelium and mesenchyme. The mesenchyme expresses AR and acquires the ability to induce cytological and epithelial differentiation of the prostate, which occurs in parallel with the development of the stroma [77]. From this perspective, two studies analyzed prostate morphogenesis and lesions after exposure to DBP. In the first study, Peixoto et al. verified whether exposure to DBP could increase the incidence of prostate lesions [78]. For that, a carcinogenic protocol was used that consisted of exposing female Wistar rats to 100 mg/kg of DBP with MNU (carcinogenic agent N-methyl-N-nitrosourea) or 500 mg/kg of DBP with MNU, from GD15 to PND21, then the offspring were exposed to a single intraperitoneal injection of the MNU in the sixth postnatal week (PND42), that is, after weaning, when an increase in epithelial proliferation occurs. After the administration of MNU, exogenous testosterone was administered subcutaneously twice a week for 24 weeks and suspended 15 days before euthanasia [78]. Regarding the second study, Mello Santos et al. analyzed the effects of DBP on prostate development using the same species of rat, DBP concentration and exposure period as in the study carried out by Peixoto et al. [78]; however, they did not use the carcinogenic protocol [79].

Both studies observed a decrease in anogenital distance at PND1 in offspring; however, in the study by Peixoto et al., only the group exposed to 500 mg of DBP showed statistically significant results [78,79]. Regarding serum testosterone levels, while de Mello Santos et al. observed a decrease in offspring on PND1, especially in the group exposed to 500 mg of DBP [79], Peixoto et al. found that there was no variation between the groups on PND220 (euthanasia day), demonstrating that endogenous endocrine control was not affected by the treatment, since testosterone values were restored after the suspension of exogenous testosterone administration [78]. Furthermore, this study also showed an increase in the epithelial compartment of the ventral prostate and in the incidence of inflammation and metaplasia/dysplasia in the dorsolateral lobe in both groups exposed to DBP. Besides this, animals treated with 500 mg of DBP demonstrated an increased incidence of prostatic intraepithelial neoplasia. The proliferation index of ventral prostate epithelial cells increased in all MNU-treated groups; however, this increase was only statistically significant in the group exposed to 500 mg of DBP. When analyzing gene expression, the group exposed to 100 mg of DBP had an increased expression of AR and MAPK (ERK1/2), while the group exposed to 500 mg of DBP had higher expressions of ERα and AKT. Overall, Peixoto et al. concluded that perinatal exposure to DBP may increase the likelihood of adult rats developing prostate lesions [78]. In the study conducted by de Mello Santos et al., it was found that, on PND1, exposure to 500 mg of DBP decreased the number and length of prostate buds and reduced the expression of AR and BMP-4 (regulated by androgens and expressed in both epithelial and mesenchymal cells), suggesting that exposure to 500 mg of DBP may delay the formation of prostate buds. The thickness of the smooth muscle layer was also reduced, which indicates a delay in the differentiation of smooth muscle cells, interfering with the paracrine epithelial–stromal interaction and, consequently, delaying the development of epithelial cells. However, the expression of the p63 protein, expressed by basal cells and important in regulating the development and differentiation of epithelial cells, showed no differences between the groups, demonstrating that basal cells can survive with a low concentration of androgens [79]. Besides this, on PND21, the group exposed to 500 mg of DBP maintained an obvious reduction in the thickness of the smooth muscle layer. In both groups, there was a decrease in AR expression, contrary to the results obtained by Peixoto et al. [78]. The authors concluded that prenatal exposure to DBP interferes with prostate morphogenesis in male offspring [79].

The incidence of reproductive disorders in newborns, such as cryptorchidism, and in young adult males, such as low sperm count, has been increasing over the years. Thus, these diseases may represent a testicular dysgenesis syndrome originating from fetal life [80]. Therefore, twelve studies were carried out regarding testicular disorders after exposure to DBP.

In 2017, Shen et al. analyzed the effects of prenatal exposure to DBP on testicular germ cell apoptosis, while Ma et al. studied testicular development after exposure to this phthalate [81,82]. In both studies, pregnant female Sprague-Dawley rats were used and exposed to 500 mg/kg/day DBP from GD12 to GD19 [81] and to 50, 250 or 500 mg/kg/day from GD12.5 to GD21.5 [82]. Shen et al. observed that after exposure to DBP, the position of the testicles was higher on both GD19.5 and PND45 and the incidence of hypospadias and cryptorchidism at PND45 was 37.5% and 65.6%, respectively, while the incidence of both simultaneously was 31.3% [81]. On the other hand, Ma et al. observed that, at PND90, male offspring showed reduced anogenital distance [82].

In the histological assays of the testis, Shen et al. observed at GD19.5 increased cellular vacuolation, the breakdown of the mitochondrial crista, the degeneration of vacuoles in the cytoplasm and the presence of multinucleated gonocytes. At PND45, there was also an increase in cellular vacuolization, and the seminiferous tubules did not present sperm, only spermatogonia [81]. Similar results were found by Ma et al., where the groups treated with 250 and 500 mg/kg/day of DBP presented vacuoles in the seminiferous tubules and loss of spermatogenesis, in addition to the severe atrophy and weakening of the testicular tissue, while those treated with 50 mg/kg/day showed mild damage [82]. Besides this, Shen et al. found that in utero exposure to DBP led to the increased expression of the anti-apoptotic protein Bcl-2 in the embryonic mouse testis and inhibited germ cell apoptosis, which could have influenced the development of spermatogonia. In the testes of adolescent rats (PND45), the expression of pro-apoptotic proteins Bax and p53 increased, leading to the increased apoptosis of germ cells and Sertoli cells, culminating in spermatogenic dysfunction and eventually male infertility [81]. Similarly, Ma et al. observed that DBP inhibited cell proliferation and promoted apoptosis of cells in the seminiferous tubules from birth to PND21, and the group exposed to 500 mg/kg/day maintained these results until PND90. However, the balance between cell proliferation and apoptosis was restored with the increasing age of the offspring in the groups exposed to low and medium DBP concentrations. Furthermore, there was a negative regulation of the Rads1 gene, which could promote excessive cell proliferation through the increased phosphorylation of MEK1/2 in the groups exposed to 50 and 250 mg/kg/day of DBP at PND90 [82].

The following year, Christante et al. and Negrin et al. evaluated whether exposure to DBP (100 mg/kg/day) from GD8 to GD23 interferes with the development of the reproductive system and testicles of Mongolian gerbils [34,35]. The studies used different DBP solvents, Christante et al. evaluated the effects of mineral oil, while Negrin et al. used corn oil, a common solvent [34,35]. At the end of the first week of life, the number of gonocytes decreased and multinucleated gonocytes appeared in Mongolian gerbils exposed to DBP. Furthermore, cell proliferation in the seminiferous tubules increased, associated with an increase in testosterone concentration and a decrease in estrogen during this period. At the end of the fourth week of life, testicular changes occurred in rats exposed to DBP. Exposure to mineral oil delayed gonocyte differentiation at 14 days of life and increased plasma estrogen concentration at 28 days, which was not observed in the group exposed to DBP. This suggests that mineral oil and DBP may act through different pathways during this period. Nevertheless, both DBP and mineral oil interfered with the testicular development and plasma concentrations of testosterone and estradiol, which was more pronounced in the first week of life of Mongolian gerbils [34]. On the other hand, the maternal ingestion of a small amount of corn oil (100 μL/day) led to an increase in serum estradiol, similar to that observed with mineral oil by Christante et al. [34], as well as the expression of ERα in the testes of male offspring [35]. Furthermore, there was also a decrease in sperm reserve and an increase in seminiferous tubules with premature cell detachment. These results contrast with those obtained by Lourenco et al. [74], who concluded that corn oil did not affect testicular toxicity. Besides this, gestational exposure to 100 mg/kg/day of DBP diluted in 100 μL of corn oil further reduced sperm reserve, impaired sperm motility and increased serum and testicular estradiol levels, without altering testosterone levels [35]. This increase in the intratesticular concentration of estradiol may be responsible for the increase in apoptotic cells in the testis. Furthermore, serum CAT and SOD activity was increased in both the corn oil-exposed group and the DBP-exposed group. With these results, Negrin et al. concluded that both corn oil and DBP altered circulating estradiol levels, suggesting that the vehicle can potentiate the effects of DBP [35].

From a different perspective, Rashad et al. exposed pregnant female albino Wistar rats to 500 mg/kg/day of DBP from GD12 to PND14, to evaluate the apoptosis and growth of testicular cells [83]. There was a decrease in testicular weight and serum testosterone concentration in both pre-puberty (PND25) and post-puberty (PND60). The c-Myc gene, which regulates cell proliferation and apoptosis and represses the BRD7 and GAS1 genes, important in the process of cell cycle arrest, showed hypermethylation in the promoter and a consequent decrease in its expression in pre-puberty, together with a reduction in the expression of genes related to apoptosis (p53 and Bax) and an increase in the anti-apoptotic gene Bcl-2. However, in post-puberty, these genetic changes were not observed. The group exposed to DBP showed an increase in the expression of the BRD7 and GAS1 genes, probably due to epigenetic changes in the promoter of the c-Myc gene. In the testes of rats exposed to DBP, necrosis and desquamation of the seminiferous tubules of spermatogonia occurred in pre-puberty, while in post-puberty there was a marked degeneration and necrosis of spermatogonia. Rashad et al. concluded that exposure to DBP causes hypermethylation of the c-Myc gene promoter, leading to the dysregulation of genes regulating apoptosis and cell cycle arrest, resulting in reproductive dysfunction [83].

A testicle that is not typically found at the base of the scrotum is referred to as cryptorchidism, also known as an undescended testicle or retinal testicle. One or both sides may be affected by cryptorchidism. The testis may be located in the belly, inguinal region, suprascrotal region, or high scrotal region along its typical descent path from the abdomen to the scrotum [84]. Orchidopexy is a surgical procedure to reposition the testicles in the scrotum. It should be performed between 6 and 18 months of age to preserve fertility and prevent the development of malignancy in children with cryptorchidism [85]. Two studies form the same research group used pregnant female Sprague-Dawley rats and exposed them to 500 mg/kg/day of DBP [86,87]. In the first study, the animals were exposed from GD12 until PND21, when weaning occurred, to study the effects on male offspring [86], while the other exposed the animals from GD12 to GD21, and the offspring continued to be exposed through lactation and later through the diet with 6000 ppm DBP [87]. The first study carried out three experiments, as follows [87]: experiment 1 evaluated adult testes on PND112 after exposure to DBP; experiment 2a investigated the morphological changes of adult testes after the surgical induction of cryptorchidism on PND21, followed by orchidopexy on PND42; experiment 2b evaluated whether the induction of cryptorchidism increased testicular susceptibility to DBP, even after orchidopexy [87].

Regarding the results, in experiment 1, on PND4, the offspring showed a decrease in anogenital distance, while in experiment 2b, there were no significant differences in anogenital distance after exposure to DBP [87]. Comparing with the second study, a decrease in body weight and anogenital distance was demonstrated, as well as an increase in the frequency of nipple retention [86]. Still in experiment 2b, on PND21, the testicles of 2 of the 10 animals exposed to DBP were poorly positioned, being corrected before the surgical induction of cryptorchidism, and on PND112, all animals exposed to DBP had testicles with reduced weight, which was not statistically significant in the animals of experiment 1 [87]. In contrast, Souza et al. only observed a reduction in testicular weight in pre-puberty (PND24), with no changes in puberty (PND45) [86]. In experiment 1, in PND112, the seminiferous tubules had a normal appearance, although some contained only Sertoli cells [87]. In experiment 2a, the weight of the testicles of animals with induced cryptorchidism decreased, with the development of atrophy of the germinal epithelium and the absence of sperm in the tubular lumen, in addition to apoptotic bodies and multinucleated germ cells [87]. After orchidopexy, there was almost complete recovery of the structure of the testicles, despite the continued reduction in testicular weight [87]. In experiment 2b, after exposure to DBP, the testes showed disrupted spermatogenesis, intraepithelial vacuolation, and the exfoliation of germ cells with occasional intratubular calcification [87]. In this case, orchidopexy was unable to fully reverse these changes, indicating that DBP inhibited the effectiveness of surgery in reversing testicular injuries caused by cryptorchidism [87]. The authors concluded that isolated exposure to DBP did not cause severe testicular lesions, unlike cryptorchidism, which induced more severe lesions; however, these were completely reversed by orchidopexy. Furthermore, when rats were simultaneously exposed to DBP and cryptorchidism, surgery was not effective in reversing testicular lesions, possibly due to additive or synergistic interactions between the mechanisms of DBP and cryptorchidism [87]. In the second study, the authors observed a reduction in the expression of the Pou5f1 and Mki67 genes at PND24, which are important for the regulation of cellular pluripotency and the occurrence of mitosis, respectively [86]. At PND45, there was a reduction in the expression of Pou5f1 and Spry, the latter being involved in several cellular functions, including anti-proliferative action, the inhibition of cell migration, and the promotion of differentiation and survival. Thus, exposure to DBP affected spermatogonia by reducing pluripotency and cell differentiation potential [86].

The tight junctions between Sertoli cells are crucial for the blood–testicular barrier that protects germ cells. These junctions are formed by transmembrane proteins, occludins and claudins, and can be regulated by NF-κB/COX-2/prostaglandin E2 (PEG2), being degraded by the metalloproteinases of extracellular matrix (MMPs) [88]. In three studies conducted by the same research group, pregnant female BALB/c mice were exposed to 50, 250 and 500 mg/kg/day of DBP, from GD12.5 to GD21.5, to investigate DBP’s effects on tight junction formation during testicular development, the impact of DBP on cell proliferation and apoptosis in male offspring, and the effects on the structure and function of the testicles in adulthood [89,90,91].

The results show that the groups treated with 250 or 500 mg/kg/day of DBP had immature germ cells, increased cellular disarray, disassembled tight junctions and vacuoles. O the other hand, the group exposed to 50 mg/kg/day of DBP presented an increase in actin filaments, which may be related to the advanced formation of tight junctions, greater testicular development, and the formation of the blood–testicular barrier at PND22 [90]. Additionally, there was an increase in the proliferation of Sertoli cells in the testicles [89]. An increase in the expression of the occludin protein in the group exposed to 500 mg of DBP was also observed, and opposite to the 50 mg of DBP exposure group, those exposed to 250 and 500 mg showed an increase in the expression of MMP-2, MMP-9, MMP-8, Act1, p65 and COX-2 proteins. Thus, the authors concluded that maternal exposure to DBP promotes the formation of testicular tight junctions through the negative regulation of NF-κB/COX-2/PEG2/MMP-2, which may favor the development of the testicles during puberty [90]. Furthermore, exposure to a lower concentration of DBP accelerated the formation of the blood–testicular barrier in rats, explaining the precocious puberty observed in the most recent study by Ma et al. [90,91]. Besides this, Ma et al. found that the group exposed to 50 mg/kg/day of DBP showed a reduced expression of Peli2, a ubiquitin ligase crucial in the ubiquitination and degradation of interleukin-1 receptor-related kinase 1 (IRAK1), leading to the activation of the MAPK/JNK signaling pathways, associated with cell proliferation, migration and regeneration. Furthermore, in this group, c-Jun phosphorylation in the testes also increased, indicating that DBP promoted cell proliferation through the MAPK/JNK signaling pathway [89]. In their 2021 study, Ma et al. observed a decrease in anogenital distance and testicular weight. At the beginning of puberty (PND22), serum levels of GnRH, LH, FSH and testosterone were elevated, stimulating morphological changes in the testicles, and on PND50, these hormones were reduced, but increased again on PND90, likely due to the negative feedback mechanism. Thus, exposure to DBP leads to the onset of precocious male puberty in rats, probably due to increased serum levels of LH, FSH and testosterone [91].

From a different perspective, van den Driesche et al. investigated the effects of exposure to 750 mg/kg/day of DBP in Wistar rats, during the MPW, specifically from E15.5 to the E18.5. Male offspring were studied on E17.5 (within the MPW), E21.5 (late gestation) or PND90 (adulthood) [92]. On the other hand, Li et al. evaluated whether taxifolin, a flavonoid with anti-proliferative, antioxidant and anti-inflammatory effects, could reverse toxicity in male offspring. For this, female Sprague-Dawley rats were exposed from GD12 to GD21 and divided into six groups: Group 1, control exposed only to corn oil; Group 2, exposed daily to 10 mg/kg of taxifolin; Group 3, exposed daily to 20 mg/kg of taxifolin; Group 4, exposed daily to 500 mg/kg of DBP; Group 5, exposed daily to 500 mg/kg of DBP and 10 mg/kg of taxifolin; and Group 6, exposed daily to 500 mg/kg of DBP and 20 mg/kg of taxifolin [93].

In the van den Driesche et al. study, exposure to DBP during MPW led, in adult male rats, to a decrease in anogenital distance, anogenital index, mean testicular weight, penis length and weight, and the weight of the ventral prostate and the seminal vesicle [92]. Li et al. also observed a decrease in anogenital distance due to the reduction in testosterone, resulting from the decrease in the expression of the genes and proteins Cyp11a1, Hsd17b3 and Insl3. When supplemented with taxifolin, these effects were reversed in both group 5 and group 6 [93]. Van den Driesche et al. also found a reduction in sperm motility and count, a decrease in testosterone and an increase in LH on E17.5, which remained to a lesser extent until E21.5. On E17.5, there was a decrease in the mRNA expression of the StAR, Cyp11a1 and Cyp17a1 genes, which returned to control levels on E21.5. Furthermore, 81% of exposed rats showed cryptorchidism and 23% hypospadias [92]. Both studies showed that exposure to DBP resulted in a reduction in small aggregates of fetal Leydig cells and an increase in large aggregates, indicating the development of testicular dysgenesis [92,93]. Once again, the administration of taxifolin reversed these DBP-induced adverse effects in fetal Leydig cells [93]. Besides this, DBP did not affect the number of Sertoli cells, but downregulated SOX9 expression in these cells, with only the highest concentration of taxifolin being able to reverse these effects. DBP also reduced the expression of the gene and protein Nr5a1 (nuclear receptor group A1 subfamily 5), a transcription factor crucial for the development of fetal Leydig cells and Sertoli cells. However, in this case, taxifolin was unable to reverse this effect. DBP reduced the expression of the SOD1 (superoxide dismutase 1) and GPx1 genes and increased MDA levels, indicating a development of lipid peroxidation and a decrease in endogenous antioxidants, which was reversed by taxifolin supplementation. There were also decreases in the levels of SIRT1/PGC-1α (a class III histone deacetylase that targets PGC-1α, both present in fetal Leydig cells and Sertoli cells), the phosphorylation of AMPK (an important metabolic sensor), and Bcl2, without changes in the level of Bax protein [93]. Overall, Li et al. concluded that DBP can lead to the development of testicular dysgenesis syndrome through increased ROS generation and the negative regulation of the antioxidant system via the SIRT1/PGC-1α pathway [93]. The authors also suggested that taxifolin may be an effective antioxidant for reversing the adverse effects of DBP on fetal testes [93].

Phthalates exposure has also been investigated in the development of erectile dysfunction. Erectile function is modulated by parasympathetic nerve terminals and is mediated by the relaxation and contraction of the smooth muscle of the penis. The eNOS and nNOS isoenzymes produce nitric oxide (NO), which, in the active state, promotes the formation of cyclic guanosine monophosphate (cGMP), which leads to the relaxation of the smooth muscle of the corpus cavernosum of the penis, resulting in erection. Erectile dysfunction is characterized by the persistent inability to achieve or maintain an erection [94]. Thus, regarding the effects of DBP, there is only one study analyzing the effects of its exposure on erectile function, using pregnant female Sprague-Dawley rats exposed to 12.5, 100 or 800 mg/kg/day of DBP, from GD13 to GD21 [95]. The results show that maternal exposure to DBP increased the expression of TGF-β1 and decreased the expression of the α-SMA in the corpus cavernosum. All groups showed testicular damage with a decrease in germ cells and serum testosterone levels, leading to a reduction in the penis weight and the thickness of the myelin sheath in the right cavernous nerves. Body weight increased significantly in the medium dose group. Furthermore, exposure to DBP can cause endothelial dysfunction, decreasing eNOS and nNOS expression, thus impairing the rats’ erectile function. Thus, Zhou et al. concluded that maternal exposure to DBP caused penile fibrosis, and decreased testosterone levels and endothelial dysfunction, attributed to the activation of the Akt/Bad/Bax/caspase-3 pathway and suppression of the NOS/cGMP pathway in the penis, leading to erectile dysfunction [95].

Regarding the effect of DBP on the duration of the spermatogenesis cycle, Wakui et al. studied how prenatal exposure to this EDC affects adolescent (9 weeks of age) and adult (17 weeks of age) rats. For this, the authors used pregnant female Sprague-Dawley rats exposed to 10, 50 or 100 mg/kg/day of DBP from GD12 to GD21 [96]. At both 9 and 17 weeks of age, decreased testicular testosterone levels and increased serum FSH levels were observed. In the group exposed to 100 mg/kg of DBP, there was a delay in the time of spermatogenesis cycle (16.95 ± 0.87 h at 9 weeks of age and 19.01 ± 1.07 h at 17 weeks of age) due to the increase in spermatocytes in stages VIII and IX and a decrease in spermatocytes in stages VII and X. In addition, there were also abnormalities in Sertoli cells, contributing to deficient testicular spermatogenesis. In summary, the authors concluded that there is greater delay and impairment to spermatogenesis in adult rats than in adolescent rats [96].

In summary, prenatal exposure to DBP has been consistently associated with several impairments in the development of the male reproductive system in in vivo studies. Evidence indicates that exposure to DBP reduces anogenital distance and reduces the weight of the testicles. Furthermore, this phthalate causes a decrease in testosterone and an increase in LH and FSH levels, possibly due to the downregulation of important genes in steroidogenesis, such as StAR, Cyp11a1, Hsd3b, Cyp17a1 and Hsd17b. Consequently, Leydig cell hyperplasia was observed, along with deficiency in spermatogenesis and the presence of multinucleated gonocytes in the testes of offspring prenatally exposed to DBP. These results suggest that prenatal exposure to DBP may be related to reproductive dysfunction and male infertility in adulthood.

### 3.6. Female Reproductive System

In contrast to the male reproductive system, the female reproductive system has been less widely studied. The estrous cycle of animals has several phases with distinct cytological characteristics: the proestrus phase, characterized by the presence of nucleated epithelial cells; the estrus phase, with a predominance of cornified/anucleated epithelial cells; the metestrus phase, with a predominance of cornified/anucleated, nucleated epithelial cells and leukocytes; and the diestrus phase, characterized by the presence of leukocytes [97]. Thus, there are two studies wherein the effects of DBP have been analyzed in female Sprague-Dawley rats, and there is one study wherein this effect has been analyzed in the CD1 species of mice.

Xie et al. exposed female Sprague-Dawley rats to 10, 100 or 600 mg/kg/day of DBP, from GD12 to PND21, with the aim of examining the reproductive toxicity of maternal exposure to DBP [97]. On the other hand, Zhang et al. analyzed whether perinatal exposure to DBP caused ovarian dysfunction through the dysregulation of ER signaling and the TGF-β signaling pathway, an ER-dependent cytokine that regulates cell growth, differentiation and apoptosis. For this, the animals were exposed to 33, 66 and 132 mg/kg/day of DBP, from GD7 until PND21 [98]. In the first study, the group exposed to 10 mg/kg/day of DBP showed increased serum levels of estradiol in the proestrus, diestrus and metestrus phases, and increased serum progesterone levels in all phases of the estrous cycle, at PND63. In the groups exposed to 100 and 600 mg/kg/day of DBP, there was only an increase in estradiol and progesterone levels in the proestrus phase. The authors suggest that this hormonal increase may reduce egg quality and libido in rats, as well as increasing the risk of female toxicity [97]. On the other hand, Zhang and co-workers found that exposure to DBP increased the abortion rate, decreased the number of offspring per litter, and increased female offspring weight and ovary weight. The estrous cycle was prolonged in a dose-dependent manner, probably due to the increase in the proliferation of granulosa cells and the number of secondary follicles. However, the mRNA and protein levels of TGF-β2, TGF-β3 and TGF-βRII, as well as the mRNA levels of LH and FSH receptors and CYP19a, decreased after exposure to DBP. These results allowed the authors to conclude that maternal exposure to DBP alters the genetic expression of TGF-β signaling and important functional proteins in the ovaries of adult female offspring [98].

In CD1 mice, germ cells begin meiosis 13.5 days after coitus and go through several stages of meiotic prophase I. This process begins with numerous programmed double-stranded DNA cuts, which are repaired by homologous recombination, resulting in the production of healthy oocytes [99]. Thus, Ma et al. [100] used pregnant CD1 mice, exposed to 10 or 50 mg/kg/day of DBP from the 14.5th day after coitus over three days, or until the day of euthanasia. The authors observed that the maternal exposure to 10 mg/kg of DBP resulted in a decrease in primordial follicles, but accelerated follicular development, increasing the number of secondary follicles, consistent with the results of Zhang et al. [98,100]. Furthermore, exposure to 10 mg/kg/day of DBP delayed meiotic progression and induced DNA damage [100]. The foci of DMC1, RAD51 and MLH1, essential for homologous recombination, were reduced, although double-stranded DNA cuts were repaired efficiently. However, the K544cr levels of MSH6 decreased, impairing MSH6 binding to the ku70 protein and increasing the latter’s levels, which led to the repair of DNA nicks by non-homologous end-joining rather than homologous recombination. In short, gestational exposure to DBP caused changes in maternal behavior in adulthood, such as increased anxiety, inhibited locomotor activity and changes in neonatal care, potentially impacting the development of offspring. Furthermore, exposure to DBP resulted in precocious puberty and birth weight gain in the F2 generation [100].

In summary, exposure to DBP increased serum estradiol and progesterone levels, suggesting potential negative impacts on egg quality and libido in rats. There was also a higher miscarriage rate, along with increased ovarian weight and genetic alterations in TGF-β signaling, in the ovaries of adult female offspring. The number of secondary follicles increased, with DBP slowing the meiotic progression and inducing DNA damage, with the repair of DNA nicks occurring by joining non-homologous ends.

**Table 2:** Summary of DBP’s effects from the in vivo studies on male and female reproductive systems.

System	Cells Organ	Reference	Animal	DBP Dose	Solvent	Route of Exposure	Duration of Exposure	Main Conclusions
Male reproductive system	Leydig cells	Wakui et al., 2013 [38]	Sprague-Dawley rats	100 mg/kg/day	Corn oil	Intragastrically	From GD12 to GD21	**1**. Decreased size and weight of the offspring’s testicles **2**. Leydig cells with hyperplastic lesions and reduced smooth endoplasmic reticulum, possibly explained by the low testosterone levels **3**. No microbodies observed in hyperplastic Leydig cells
		Wakui et al., 2014 [37]	Sprague-Dawley rats	100 mg/kg/day	Corn oil	Intragastrically	From GD12 to GD21	**1**. Elevated LH levels due to a compensatory reaction to decreased testosterone **2**. Leydig cell hyperplasia, possibly due to increased LH levels, low testosterone levels, and high ERα expression in these cells
		Motohashi et al., 2016 [64]	Sprague-Dawley rats	100 mg/kg/day	Corn oil	Intragastrically	From GD12 to GD21	**1.** Decreased testicular testosterone during the juvenile and peri-puberty periods are due to the downregulation of StAR and Cyp11a1 **2**. After peri-puberty, low testicular testosterone levels due to downregulation of Hsd3b, Cyp17a1 and Hsd17b3
		Chen et al., 2017 [39]	Sprague-Dawley rats	100, 500 mg/kg/day	Corn oil	Oral	From GD12 to GD21	**1**. Decreased serum testosterone levels **2.** At PND7, an increased number of fetal Leydig cells, more pronounced at PND14 and PND21 **3.** Delay in Leydig cell development due to fewer immature Leydig cells at PND28 and adult Leydig cells at PND56 **4**. Downregulation of GnRH contributed to lower LH expression and delayed Leydig cell development
	Sertoli cells	Okayama et al., 2017 [40]	Sprague-Dawley rats	100 mg/kg/day	Corn oil	Oral	From GD12 to GD21	**1**. At weeks 14 and 17 of age, male offspring prenatally exposed to DBP showed testicular atrophy characterized by smaller testes and reduced-diameter seminiferous tubules **2**. Increased number of Sertoli cells and their proliferation from puberty to adulthood, with a decrease in testicular testosterone and an increase in serum FSH
		Lara et al., 2017 [68]	Rats	750 mg/kg/day	Corn oil	Oral	FW, from E13.5 to E20.5; MPW, from E15.5 to E18.5; LW, from E19.5to E20.5	**1**. Increased number of germ cells migrating to the basal lamina upon DBP exposure during MPW leading to rupture of the weakened basal lamina
		Xie et al., 2022 [71]	Sprague-Dawley rats	750 mg/kg/day	Corn oil	Oral	From GD14 to GD18	**1**. Increased RANKL expression and decreased spermatogonia in the seminiferous tubules **2**. After exposure to DBP, RANKL expression in Sertoli cells increased through the NF-κB/COX-2 signaling pathway, resulting in spermatogonia apoptosis
	Hormones	Pike et al., 2014 [70]	Sprague-Dawley rats	100, 500 mg/kg/day	Corn oil	Oral	From GD16 to GD20	**1.** Enlargement of seminiferous tubules with multinucleated gonocytes **2**. Exposure to 500 mg DBP decreased testicular testosterone levels and male anogenital distance at GD20 and PND5 **3.** Marcks, Pum1, Penk and Nupr1 may be postnatal markers of prenatal DBP exposure
		Giribabu et al., 2014 [73]	Albino Wistar rats	100, 500 mg/kg/day	Corn oil	Oral	On GD1, GD7, GD14	**1.** Decreased sperm, sperm motility and viability, and presence of morphologically altered sperm **2.** Decreased activity of 3β-HSD1 and 17β-HSD3 enzymes and serum testosterone levels, and increased FSH and LH levels in the testes of adult rats **3**. Reduced reproductive performance in adulthood
		Lourenco et al., 2014 [74]	Wistar rats	500 mg/kg/day	Corn oil, canola oil and fish oil	Oral	From GD13 to GD20	**1.** Decreased anogenital distance and testicular testosterone levels in offspring, regardless of the vehicle **2**. All groups presented multinucleated gonocytes in the seminiferous tubules, but exposure to DBP increased this occurrence and the diameter of the tubules **3**. Increased large clusters of Leydig cells and decreased small clusters **4**. Vehicles did not contribute to the variability of results
		Li et al., 2015 [47]	Wistar rats	100, 300, 900 mg/kg/day	Corn oil	Intragastrically	From E12.5 to E20.5	**1**. Exposure to lower concentrations of DBP may cause a reversible delay in testicular development **2**. Exposure to 900 mg of DBP can induce irreversible testicular damage **3**. Administration of exogenous testosterone normalized testosterone levels and increased anogenital distance
		Zhu et al., 2016 [48]	Sprague-Dawley rats	850 mg/kg/day	Corn oil	Gastric intubation	From GD11 to GD15	**1**. Decreased anogenital distance, testicular size and serum testosterone concentration **2**. Presence of Leydig cells with hyperplasia and an abnormally shaped lumen
		Giribabu et al., 2017 [75]	Wistar rats	100, 500 mg/kg/day	Corn oil	Intra-peritoneal injection	On GD1, GD7, GD14	**1**. DBP induces reproductive toxicity in male rats exposed prenatally, i.e., decreased fertility, testicular and seminal vesicle weight, serum testosterone and increased oxidative stress **2.** Testosterone supplementation antagonized reproductive toxicity caused by DBP
	Multinucleated germ cells	Spade et al., 2015 [76]	Sprague-Dawley rats	500 mg/kg	Corn oil	Oral	From GD17 or GD18. First dose at 0 h and second at 24 h	**1**. Exposure to DBP altered the morphology of the seminiferous tubules in the first 24 h after initial exposure, when treatment started on GD18 **2**. Increased diameter of the seminiferous tubules and the rate of MNGs after 24–48 h of exposure to DBP **3**. Exposure starting on GD18 increased cell mortality within 48 h **4**. MNGs formed through a mechanism not dependent on the proliferation of germ cells, since exposure to DBP did not alter cell proliferation
	Prostate	Peixoto et al., 2016 [78]	Wistar rats	100, 500 mg/kg/day	Corn oil	Oral	From GD15 to PND21	**1**. Anogenital distance at PND1 decreased in male offspring **2.** No variation of serum testosterone levels between groups on PND220 (day of euthanasia), demonstrating that endogenous endocrine control was not affected by treatment **3**. Increased epithelial compartment of the ventral prostate and incidence of inflammation and metaplasia/dysplasia in the dorsolateral lobe **4**. Increased incidence of prostatic intraepithelial neoplasia in animals treated with 500 mg of DBP **5**. Increased proliferation index of ventral prostate epithelial cells in all groups **6**. Perinatal exposure to DBP may increase the likelihood of adult rats developing prostate lesions
		de Mello Santos et al., 2017 [79]	Wistar rats	100, 500 mg/kg/day	Corn oil	Oral	From GD15 to PND21	**1**. Decreased anogenital distance and serum testosterone levels **2**. In the group exposed to 500 mg of DBP, the number and length of prostate buds were lower and there was a decrease in the expression of the androgen receptor and the BMP-4 protein and the thickness of the smooth muscle layer. Thus, DBP may delay the formation of prostate buds
	Testicles	Shen et al., 2017 [81]	Sprague-Dawley rats	500 mg/kg/day	Olive oil	Oral	From GD12 to GD19	**1**. Higher position of the testicles in the DBP-exposed group **2**. The incidences of hypospadias and cryptorchidism at PND45 were 37.5% and 65.6%, respectively, while of both simultaneously it was 31.3% **3**. Increased expression of BcI-2 protein in the embryonic mouse testis and inhibition of germ cell apoptosis **4**. Increased expressions of the pro-apoptotic proteins Bax and p53 in the testes of pubertal rats, leading to the increased apoptosis of germ cells and Sertoli cells
		Ma et al., 2017 [82]	Sprague-Dawley rats	50, 250 or 500 mg/kg/day	Corn oil	Oral	From GD12.5 to GD21.5	**1**. Decreased anogenital distance at PND90 **2**. In the group treated with 50 mg of DBP, there was slight damage to the testicular tissue. In the other groups, there was evident damage to the testicular tissue **3**. DBP inhibited cell proliferation and promoted apoptosis of cells in the seminiferous tubules. However, only the group exposed to 500 mg DBP maintained these results until PND90 **4**. Decreased Rads1 gene, promoting excessive cell proliferation through increased phosphorylation of MEK1/2 in groups exposed to 50 and 250 mg of DBP at PND90
		Christante et al., 2018 [34]	Mongolian gerbil	100 mg/kg/day	Mineral oil	Oral	From GD8 to GD23	**1**.Both DBP and mineral oil interfered with the development of the testicles and plasma concentrations of testosterone and estradiol, more pronounced in the first week of life
		Negrin et al., 2018 [35]	Mongolian gerbil	100 mg/kg/day	Mineral oil	Oral	From GD8 to GD23	**1**. Both corn oil and DBP altered lipid metabolism and circulating estradiol levels, suggesting that the vehicle may potentiate the effects of DBP
		Rashad et al., 2018 [83]	Albino Wistar rats	500 mg/kg/day	Mineral oil	Oral	From GD12 to PND14	**1**. Exposure to DBP causes the hypermethylation of the c-Myc gene promoter, leading to the dysregulation of genes that regulate apoptosis and cell cycle arrest, resulting in reproductive dysfunction in pre-puberty
		de Souza et al., 2019 [87]	Sprague-Dawley rats	500 mg/kg and 6000 pm	Corn oil	Oral and diet	From GD12 to GD21 and during the rest of the experiment through diet	**1**. Isolated exposure to DBP did not cause severe testicular lesions **2**. Cryptorchidism induced more severe lesions, which were completely reversed by orchidopexy **3**. In rats exposed to DBP and cryptorchidism, surgery was not effective in reversing testicular lesions, probably due to additive or synergistic interactions between the mechanisms of BPD and cryptorchidism
		Souza et al., 2019 [86]	Sprague-Dawley rats	500 mg/kg/day	Corn oil	Oral	From GD12 to PND21	**1**. Reduced Pou5f1 and Mki67 gene expression at PND24 and reduced Pou5f1 and Spry expression at PND45, suggesting that exposure to DBP affected spermatogonia by reducing pluripotency and cell differentiation potential
		Ma et al., 2020 [90]	BALB/c Mice	50, 250, 500 mg/kg/day	Corn oil	Oral	From GD12.5 to GD21.5	**1**. DBP 250 or 500 mg/kg/day groups showed immature germ cells, increased cell disarray, disassembled tight junctions and vacuoles **2**. DBP 50 mg group had an increase in actin filaments, and promoted testicular development and formation of the blood–testicular barrier **3.** Maternal exposure to DBP promotes the formation of testicular tight junctions through the negative regulation of NF-κB/COX-2/PEG2/MMP-2, which may favor testicular development during puberty
		Ma et al., 2020 [89]	BALB/c Mice	50, 250, 500 mg/kg/day	Corn oil	Oral	From GD12 to birth	**1**. Offspring prenatally exposed to 50 mg of DBP showed increased proliferation of Sertoli cells in the testes **2**. The group exposed to 50 mg of DBP showed a reduction in Peli2 expression, leading to the activation of the MAPK/JNK **3**. A signaling pathway, c-Jun phosphorylation, in the testes after exposure to 50 mg DBP increased, indicating that DBP promoted cell proliferation through the MAPK/JNK signaling pathway
		van den Driesche et al., 2020 [92]	Wistar rats	750 mg/kg/day	Corn oil	Oral	From E15.5 to E18.5	**1**. Exposure to DBP during MPW led, in adult males, to a decrease in anogenital distance, anogenital index, mean testicular weight, penis length and weight, ventral prostate and seminal vesicle weight **2**. Reduction in motility and sperm count, decreased testosterone and increased LH **3**. Here, 81% of the rats presented cryptorchidism and 23% hypospadias **4**. On E17.5, they found a decrease in the expression of steroidogenic synthesis genes; however, on E21.5, this reduction returned to control levels **5**. Reduction of small aggregates of fetal Leydig cells and increase of large aggregates, indicating the development of testicular dysgenesis
		Li, Yu, et al., 2020 [93]	Sprague-Dawley rats	500 mg/kg/day	Corn oil	Oral	From GD12 to GD21	**1.** Increased multinucleated gonocytes in the seminiferous tubules, reversed by taxifolin **2**. Reduction in small aggregates of fetal Leydig cells and increase in large aggregates, indicating the development of testicular dysgenesis, prevented by taxifolin **3**. Decreased anogenital distance due to reduced testosterone, resulting from the decreased expression of Cyp11a1, Hsd17b3 and Insl3. Supplementation with taxifolin reversed these effects in groups 5 and 6 **4**. DBP led to the development of lipid peroxidation and a decrease in endogenous antioxidants, reversed by taxifolin **5.** Decrease in SIRT1/PGC-1α levels, AMPK and BCl2 phosphorylation
		Ma et al., 2021 [91]	BALB/c Mice	50, 250, 500 mg/kg/day	Corn oil	Oral	From GD12 to birth	**1.** Exposure to DBP leads to precocious male puberty in rats, probably due to increased serum levels of LH, FSH and testosterone
	Spermatogenesis cycle	Wakui et al., 2022 [96]	Sprague–Dawley rats	10, 50, 100 mg/kg/day	Corn oil	Intragastrically	From GD12 to GD21	**1**. At 9 and 17 weeks of age, exposure to 100 mg DBP decreased testicular testosterone levels and increased serum FSH levels **2**. Spermatogenesis is more delayed and impaired in adult mice than in puberty
	Erectile function	Zhou et al., 2021 [95]	Sprague–Dawley rats	12.5, 100, 800 mg/kg/day	Corn oil	Gastric intubation	From GD13 to GD21	**1**. Decreased testosterone level **2**. DBP caused penile fibrosis and endothelial dysfunction, attributed to the activation of the Akt/Bad/Bax/caspase-3 pathway and suppression of the NOS/cGMP pathway in the penis, leading to erectile dysfunction
		Hunter et al., 2021 [27]	Sprague Dawley rats	500 mg/kg	Corn oil	Oral	From GD14.5 to PND6, every two days	**1**. Reduction of male anogenital distance
**Female Reproductive System**		Xie et al., 2016 [97]	Sprague Dawley rats	10, 100, 600 mg/kg/day	Corn oil	Oral	From GD12 to PND21	**1**. The lowest concentration of DBP increased the serum estradiol levels in the proestrus, diestrus and metestrus phases, and increased the serum progesterone levels in all phases of the estrous cycle **2**. The medium and high concentration of DBP increased estradiol levels and offspring progesterone only in the proestrus phase
		Zhang et al., 2022 [98]	Sprague Dawley rats	33, 66, 132 mg/kg/day	Corn oil	Intraperitoneal	From GD7 to PND21	**1**. Prolonged estrous period possibly due to increased proliferation of irregularly shaped granulosa and increased number of secondary follicles **2**. Decreased TGF-β2, TGF-β3 and TGF-βRII mRNA and protein levels, as well as levels of LH and FSH receptors and CYP19a mRNA after exposure to DBP
		Ma et al., 2023 [100]	CD1 mice	10, 50 mg/kg/day	Corn oil	Oral	From GD14.5 for 3 days or until the day of sacrifice	**1.** Decreased primordial follicles and increased number of secondary follicles **2**. DBP delayed meiotic progression and induced DNA damage, with the repair of DNA cuts occurring through non-homologous end joining

## 4. Epidemiological Studies

As far as epidemiological studies on the effects of DBP are concerned, there are only studies on the neurological and reproductive systems, which are summarized in Table 3.

### 4.1. Nervous System

Language delay is an important indicator of children’s neurological development. For this reason, a study has been carried out linking the levels of metabolites of various phthalates, particularly DBP, in urine samples in the first trimester of pregnancy and the language development of these children [101]. Two independent cohorts were used for this study, the Swedish Environmental Longitudinal Mother and Child, Asthma and Allergy (SELMA) in Sweden and The Infant Development and Environment Study (TIDES) in the United States. For the SELMA study, data were collected from 1 November 2007 to 30 June 2013; however, from 1 November 2016 to 30 June 2018, 968 mother–child pairs were analyzed, in which the first morning urine was collected at the first prenatal visit. For the TIDES study, data were collected from 1 January 2010 to 29 March 2016; however, from 15 September 2016 to 30 June 2018, 370 mother–child pairs were analyzed, where spot urine samples were collected at the first prenatal visit. The children’s language development was assessed using a screening questionnaire, wherein a vocabulary of 50 words or less was classified as language delay. In both cohorts, the authors observed that exposure to DBP in the first trimester of pregnancy seems to be associated with a delay in language development in children aged between 2.5 and 3 years, with statistically significant results only in boys [101].

In the following year, a study was carried out to analyze whether the development of neural tube defects in fetuses was associated with a high maternal amount of DBP and its metabolite MBP [21]. This study was carried out in Qian’xi, Hebei province, where 44 mothers with fetuses with neural tube defects were studied from December 2006 to December 2010. After inducing abortion, since the fetuses had spina bifida or anencephaly, 41 samples of urine and fetal tissue were collected from mother–child pairs. Serum, amniotic fluid and urine were also collected from 40 mother–child pairs to detect indicators of oxidative stress. After the maternal urinary quantification of DBP and MBP levels, the authors found detection rates of DBP and MBP in the population with neural tube defects of 48.78% and 21.95%, respectively, while in the control group, these were 12.50% and 5.00%, respectively. These results show that the incidence of neural tube defects in humans has a positive relationship with DBP. To investigate the association between neural tube defects and oxidative stress, the levels of several oxidative stress indicators, namely, SOD, MDA, glutathione (GSH) and 8-hydroxy-2-deoxyguanosine (8-OHdG), were measured in the serum, amniotic fluid and urine of women with neural tube-defective fetuses. The results reveal a significant increase in the levels of 8-OHdG, a marker of oxidative DNA damage, in amniotic fluid and urine. In addition, there was an increase in MDA levels, a marker of lipid peroxidation, in serum, amniotic fluid and urine. Conversely, the levels of SOD, an antioxidant enzyme, decreased in amniotic fluid and urine, while the GSH levels, also an antioxidant enzyme, showed no changes. These findings indicate an increase in oxidative DNA damage and lipid peroxidation, as well as a decrease in antioxidant capacity in the population with neural tube defects. Thus, Wang et al. concluded that DBP may be associated with the development of neural tube defects possibly due to oxidative stress [21].

The epidemiological studies analyzed show a significant association between exposure to phthalates, specifically DBP and its metabolite MBP, and impaired neurological development in children. It is therefore important to monitor and regulate exposure to phthalates during pregnancy, due to their potential adverse effects on children’s neurological development and the formation of the neural tube in fetuses.

### 4.2. Reproductive System

In the past, DBP was used as an insecticide and acaricide. For instance, during the Malayan Emergency (1948–1960), it was used by the New Zealand military to reduce insect and mite infestations [8]. Taking this into account, Carran et al. analyzed whether paternal exposure to DBP could lead to alterations in the development of their children. Forms were sent to servicemen in December 2009 for data collection, including deployment dates in Malaysia, use of DBP, becoming parents during their time in Malaysia or after returning to New Zealand, and reported health issues of children or grandchildren, such as the following: cryptorchidism, penile defects (e.g., hypospadias), precocious puberty (for female offspring only), low sperm count, reduced fertility, ovarian or uterine disorders and breast cancer. Besides this, the authors also verified the absorption of DBP through the military uniform, with a permeation study on cotton material representative of what was used in the uniform. Of the 71 servicemen included in the study, 58 had children after serving in Malaysia, 79 male and 76 female. The authors found that the effects of DBP on sperm were long-lasting [8]. Assuming an average body weight (bw) of 70 kg, the soldiers were exposed to approximately 64 mg/Kg bw/day of DBP. This value is close to the LOAEL (lowest observed adverse effect level) for rats, which is 50 mg/Kg bw/day, suggesting that this dose may have a relevant biological effect. The study revealed an increase in the incidence of hypospadias, breast cancer and cryptorchidism in the offspring of these soldiers. Considering the long-term high dose exposure of the soldiers to DBP, the authors proposed that this compound caused epigenetic changes in sperm DNA, which remained until conception. The increased incidence of hypospadias and cryptorchidism has been explained by the genetic modification of the zygote, which can result in an embryo with reduced testosterone synthesis, leading to poor development and growth and the feminization of the genitalia. Regarding the increased incidence of breast cancer, the authors suggested that the reduction in testosterone levels raises the estradiol/testosterone ratio, which can positively regulate the genes associated with breast cancer, thus increasing its risk [8].

The epidemiological study reviewed shows that paternal exposure to DBP can have long-lasting and adverse effects on the offspring’s reproductive system, inducing epigenetic changes in sperm DNA and leading to altered hormone synthesis and consequent abnormal development.

## 5. Knowledge Gaps and Future Perspectives

This review aims to critically analyze and discuss all the effects of prenatal exposure to dibutyl phthalate (DBP) in animal models and humans. The male reproductive system in animals has been the most widely studied topic over the years, mainly due to the so-called “phthalates syndrome”. This is a syndrome linked to the male reproductive system, with numerous abnormalities often associated with phthalates exposure, including cryptorchidism, hypopadias, and a decrease in anogenital distance [102,103]. In this system, exposure to DBP has been associated with a reduction in testosterone levels, decreased anogenital distance and the increased occurrence of multinucleated gonocytes. In addition, an impact on sperm quality and quantity has been observed, potentially leading to infertility. Regarding the effects of DBP on other systems in the human body, the studies analyzed in this review show that it may also be involved in the poor development of the offspring’s nervous system and of the male renal system by induction of hypospadias and renal fibrosis, and it may lead to anorectal malformations in male offspring and diabetes mellitus. The concentrations of DBP utilized in in vivo studies vary widely, which is advantageous given the non-monotonic dose–response relationship observed with endocrine disruptors. This characteristic indicates that increased concentrations do not necessarily result in proportionately greater effects. For example, the lowest observed adverse effect level of DBP in fetal male rats is 100 mg/kg/day [37,104], while a concentration of 750 mg/kg/day is frequently employed, as it produces a hypospadiac rat model [51,105]. Furthermore, a dosage of 66 mg/kg/day has been associated with the transgenerational inheritance of obesity following perinatal exposure to DBP [36,106]. However, there are still too few studies to be able to affirm the direct connection between this compound and the development of these diseases.

The existing knowledge gaps regarding the effects of dibutyl phthalate (DBP) on offspring following prenatal exposure primarily encompass metabolism, the nervous system, and the female reproductive system. Furthermore, the limited number of epidemiological studies raises concerns, as human responses may differ considerably from those observed in animal models. It is crucial to address these gaps in order to achieve a comprehensive understanding of the implications of DBP exposure.

Future research should focus on the consequences of maternal exposure to DBP during pregnancy and its impact on infants. Conducting prospective epidemiological studies will provide valuable insights into how exposure to DBP can have effects not only during childhood, but also into adult life. Additionally, it is essential to prioritize in vivo studies that examine the effects of longer periods of exposure. This is important because individuals are not only exposed to DBP during pregnancy, but throughout their lives. Furthermore, these in vivo studies should be conducted alongside in vitro studies to elucidate the mechanisms of action of DBP. Additionally, it may be important to conduct studies on mixtures of phthalates, as we are exposed to various endocrine disruptors. These mixtures could have different effects due to potential synergistic mechanisms that may amplify the consequences of exposure.

The results discussed in this paper emphasize the importance of the increased regulation and monitoring of phthalate exposure, particularly during pregnancy, to prevent negative impacts on fetal health and child development. It is important to prioritize the elimination of DBP from all products, particularly those intended for use by pregnant women and children, as these populations are particularly vulnerable to the potential effects of such substances.

## Figures and Tables

**Table 3:** Summary of the epidemiologic studies of DBP’s effects on the nervous and reproductive systems.

System	Reference	Population Sample	Biological Matrix	DBP Levels	Country	Cohort	Main Conclusions
Nervous	Bornehag et al., 2018 [101]	Women in the first trimester of pregnancy	Urine	SELMA: MBP levels were 69.4 ng/mL TIDES: MBP levels were 6.5 ng/mL and MiBP levels were 4.0 ng/mL	Sweden and USA	SELMA: 1 November 2007 to 30 June 2013; TIDES: 1 January 2010 to 29 March 2016	In both cohorts, exposure to DBP in the first trimester of pregnancy seems to be associated with a delay in language development in children (2.5 and 3 years old), significant in boys
	Wang et al., 2019 [21]	Mothers with fetuses with neural tube defects	Urine, fetal tissue, serum, amniotic fluid	Urine DBP levels are ≈45–55 ng/mL, and MBP levels are ≈60–70 ng/mL	China	December 2006 to December 2010	DBP may be associated with the development of neural tube defects, possibly due to oxidative stress
Reproductive	Carran and Shaw 2012 [8]	Men		Exposure dose approximately 64 mg/kg body weight/day	New Zealand	December 2009	Increased incidence of hypospadias, breast cancer and cryptorchidism in the offspring

## Data Availability

No new data were created or analyzed in this study.
